# Effects and safety of Chinese herbal medicine on inflammatory biomarkers in cardiovascular diseases: A systematic review and meta-analysis of randomized controlled trials

**DOI:** 10.3389/fcvm.2022.922497

**Published:** 2022-08-16

**Authors:** Mingdi Li, Iris Wenyu Zhou, Janine Trevillyan, Anna C. Hearps, Anthony Lin Zhang, Anthony Jaworowski

**Affiliations:** ^1^School of Health and Biomedical Sciences, RMIT University, Bundoora, VIC, Australia; ^2^Department of Infectious Diseases, Austin Hospital, Heidelberg, VIC, Australia; ^3^Life Sciences Discipline, Burnet Institute, Melbourne, VIC, Australia; ^4^Department of Infectious Diseases, The Alfred Hospital and Monash University, Melbourne, VIC, Australia

**Keywords:** inflammatory biomarkers, Chinese herbal medicine, cardiovascular disease risk, people with HIV, systematic review and meta-analysis

## Abstract

Inflammation drives cardiovascular disease (CVD) in individuals with underlying chronic inflammatory diseases, including People with HIV (PWH), independently of dyslipidemia. Adjunctive treatments that lower inflammation may be useful to lower CVD risk in such populations. There is very little data on the efficacy of Chinese herbal medicine (CHM) in reducing inflammation in PWH to address its potential in reducing this CVD risk factor, therefore we evaluated its impact on inflammatory biomarkers relevant to CVD risk in the general population. Six English and Chinese databases were searched for studies investigating CHM’s effects on inflammatory biomarkers relevant to CVD from respective inceptions to February 2022. A systematic review and meta-analysis of randomized controlled trials (RCTs) were conducted and the most-frequently prescribed herbs were identified. Thirty-eight RCTs involving 4,047 participants were included. Greater than or equal to 50% of included studies had a low risk of bias in five domains (random sequence generation, detection, attrition, reporting and other bias) and 97% had a high risk of performance bias. CHM provided significant additive effects on attenuating relevant inflammatory indices including hs-CRP (SMD −2.05, 95% CI −2.55 to −1.54), IL-6 (SMD −1.14, 95% CI −1.63 to −0.66) and TNF-α levels (SMD −0.88, 95% CI −1.35 to −0.41), but no significant effects on hs-CRP were found between CHM and placebo when co-treating with Western drugs (MD 0.04, 95% CI −1.66 to 1.74). No severe adverse events were reported in CHM groups. The two most prevalent herbs present in formulae demonstrating reduction of at least one inflammatory biomarker were Dan shen (Salviae Miltiorrhizae Radix et Rhizoma) and Huang qi (Astragali Radix). CHM, in combination with standard anti-inflammatory medications, may depress inflammation and reduce the risk of inflammatory conditions such as CVD. Rigorously-conducted trials and adequate reporting are needed to provide more robust evidence supporting the use of CHM to reduce CVD risk in people with underlying chronic inflammation such as PWH.

## Introduction

People with HIV (PWH) have an approximately twofold increased risk of cardiovascular disease (CVD) which is independent of traditional risk factors and is a major cause of morbidity and mortality in this population (1). CVD is an inflammatory disease (2) where underlying chronic inflammatory conditions are associated with heightened risk. Increased CVD risk in PWH who do not have elevated risk due to traditional risk factors is thought to be due to chronic inflammation and immune activation that persist despite virologic control with antiretroviral therapy. Persistent inflammation and innate immune activation in PWH have been suggested to result from a combination of residual human immunodeficiency virus (HIV) replication, reactivation of latent viruses such as cytomegalovirus and increased translocation of bacterial products from the gut (3). That chronic inflammation contributes significantly to increased CVD risk in PWH is evidenced by observations that high levels of inflammatory factors including hs-CRP and IL-6 are independently associated with increased risk of a cardiovascular event in PWH on effective antiretroviral therapy (4). Understanding how to reduce CVD risk due to these factors in PWH will inform strategies for other chronic conditions such as rheumatoid arthritis and cancer.

Inflammation activates monocytes and vascular endothelial cells, promoting monocyte migration into coronary arteries and initiating atherosclerotic plaque formation and progression (4). There is considerable research associating markers of monocyte activation and CVD in PWH (5) and our previous research has identified an atherosclerotic phenotype of monocytes from PWH (6) strengthening the association and suggesting plausible mechanisms for how activated monocytes may promote atherosclerosis. Decreasing inflammation in PWH receiving antiretroviral therapy may reduce the risk of age-related, non-acquired immunodeficiency syndrome (AIDS) comorbidities including CVD and alleviate excess morbidity and mortality in this population. A number of different strategies have been trialed to lower inflammation in PWH using medications aimed at targeting microbial translocation [e.g., sevelamer (3)], inhibiting innate immune signaling (7) or inhibiting cytomegalovirus (CMV) replication (8), however, many have shown limited or inconsistent efficacy in reducing HIV-related inflammation (9).

Chinese herbal medicine (CHM) as a major component of traditional Chinese medicine, is a fully institutionalized part of Chinese health care and is widely used with western medicine (9). CHM refers to both herbal formulas and single herbs: formulas contain a combination of two or more herbs prepared based on Chinese medicine theory; single herbs may be derived from plant, animal, or mineral sources and traceable in the Chinese pharmacopeia (10). In a retrospective, community-based study, it was reported that PWH who have consistently used CHM have improved lipid profiles in their blood and reduced risk of CVD (11). However, it is not known whether CHM can improve inflammatory outcomes and markers of innate immune activation, which can drive CVD in PWH independently of dyslipidemia.

As there are insufficient studies to date on the effect of CHM in reducing inflammatory biomarkers relevant to CVD in PWH, we conducted a meta-analysis of studies addressing this research question in the general population with CVD to inform future studies on PWH and provide data which may be of relevance to the potential use of CHM in other inflammatory conditions where CVD risk may be driven by factors apart from traditional risk factors.

## Materials and methods

This systematic review was conducted following the requirements of the Cochrane Handbook (12). It is reported according to the Preferred Reporting Items for Systematic Reviews and Meta-analyses (PRISMA) 2020 statement (13).

### Data sources and search strategies

Six databases (PubMed, EMBASE, Cochrane CENTRAL, CINAHL, AMED, and CNKI) were searched for peer-reviewed full-text randomized controlled trials (RCTs) published regardless of language of publication from their respective inceptions to February 2022 by using CHM, traditional Chinese medicine, CVD, inflammation/anti-inflammatory and their MeSH terms and synonyms as keywords. The results were exported into, and managed by, EndNote. The reference lists of relevant trials and reviews were screened for additional studies. The list of keywords and sample search strategies are provided in Supplementary Table 1.

### Selection criteria

Articles retrieved for evaluation were assessed by two independent reviewers. One reviewer screened the title and abstract and two independent reviewers screened the full-text of potential records for eligibility. Any disagreement was discussed between the two reviewers. If an agreement could not be reached, discrepancies were discussed and resolved with a third party.

All searched articles were screened and evaluated according to the following inclusion and exclusion criteria: articles were considered for inclusion if they (1) were RCTs, (2) recruited individuals with cardiovascular conditions (including arterial occlusive diseases, hypertension, peripheral vascular diseases, myocardial ischemia, cardiac arrest or heart arrest, CVD, ischemic heart diseases, coronary artery diseases, and atherosclerosis), (3) contained CHM in any oral forms (such as decoction and capsule) as treatment, (4) included placebo or no treatment as a control intervention and (5) assessed inflammatory biomarkers as one of the outcome measures. Trials evaluating the additive effects of CHM (i.e., CHM plus co-intervention versus co-intervention alone) were also included. Eligible co-interventions considered were western medicine (WM) drugs (for CVD and/or inflammation), lifestyle changes (e.g., dietary and exercise), and routine treatment (for CVD and/or inflammation). Routine treatment refers to three situations: (1) where only the categories of the drugs were provided (e.g., beta-blockers) but the publication did not specify drug name and/or dosage; (2) routine care such as oxygen supplementation were included in the intervention; or (3) where the authors claimed without specifying that the control group received routine/standard treatment.

Studies were excluded if they were not RCTs or recruited participants with atrial fibrillation, cerebrovascular conditions, secondary CVD (e.g., renal hypertension, pulmonary heart disease, sepsis with intraperitoneal hypertension), post-surgical conditions, underlying disease and complications (e.g., diabetes mellitus, cancers, renal diseases, cerebrovascular conditions, pulmonary conditions, periodontitis, retinal vein occlusion, erectile dysfunction, hypertensive eyeground hemorrhage, mental conditions, dementia, cardiac arrhythmia, liver cirrhosis, and hyperuricemia), or acute/emergency conditions (e.g., angina pectoris and heart failure). Studies including participants aged <18 years, or who were pregnant, were also excluded. Studies employing ineligible CHM treatment (including external application or injection), provided insufficient information on formula name/ingredients, or used inconsistent formulae in the CHM group (i.e., modified or chose formulae and varied ingredient dosage based on symptoms among participants) were excluded. Trials were also excluded if they assessed the combination of CHM and other Chinese medicine treatment (including cupping, acupuncture, electro-acupuncture, scraping, acupoint injection, bath, external CHM application etc.), employed an ineligible control group (e.g., CHM and acupuncture), failed to clearly identify the co-interventions, or assessed comparisons which could not identify the clinical effects of CHM (e.g., CHM + WM vs. placebo).

### Data extraction

All extracted data were entered into a predesigned data collection form which included details about article bibliography profile (author name, published year, language, and setting), participants (diagnostic criteria of CVD, sample size, gender, and age), interventions (number of included comparisons and duration) and outcome measures. Information about control interventions and herbal ingredients, forms and administration of treatment intervention were entered into a separate table. In the present study, outcome measures evaluated were the inflammatory biomarkers high sensitivity c-reactive protein (hs-CRP), interleukin (IL)-1, IL-6, IL-8, IL-10, monocyte chemotactic protein-1 (MCP-1), and tumor necrosis factor (TNF)-α and endothelial activation markers soluble intercellular adhesion molecule-1 (sICAM-1) and soluble vascular cell adhesion molecule-1 (sVCAM-1). The most commonly studied herbs in the included studies with positive results were summarized descriptively. Where herbal ingredients were not provided in a paper, the formula names were checked in Zhong Yi Fang Ji Da Ci Dian (14) for detailed ingredients.

### Quality assessment

The quality of included RCTs was assessed against the risk of bias (e.g., selection, performance, attrition, detection, reporting, and other biases) according to the risk of bias tool from the Cochrane Handbook (12) independently by two reviewers. Any disagreement was resolved by discussion between the two reviewers or, if required, with a third party.

### Data synthesis and analysis

The extracted quantitative data from published studies were analyzed by RevMan 5.3 (15). Continuous data were weighted by the Mean Differences (MD) with 95% confidence intervals (CI). Standardized mean difference (SMD) was used in place of MD when different scales/units were used in more than one study for the same outcome measures. Heterogeneity was assessed statistically using *I*_2_. To minimize potential heterogeneity, random effects were applied where *I*_2_ was >50% (12). Descriptive synthesis was also used to aid in data presentation when appropriate. Sensitivity analysis and publication bias were also considered where applicable. Leave-one-out sensitivity analysis and publication bias were performed where >10 studies were included. Publication bias was examined by visual inspection of funnel plots and quantified by Egger’s regression and Begg and Mazumdar’s rank correlation tests which were calculated by Meta-Essentials, version 1.4 (16).

## Results

**FIGURE 1:**
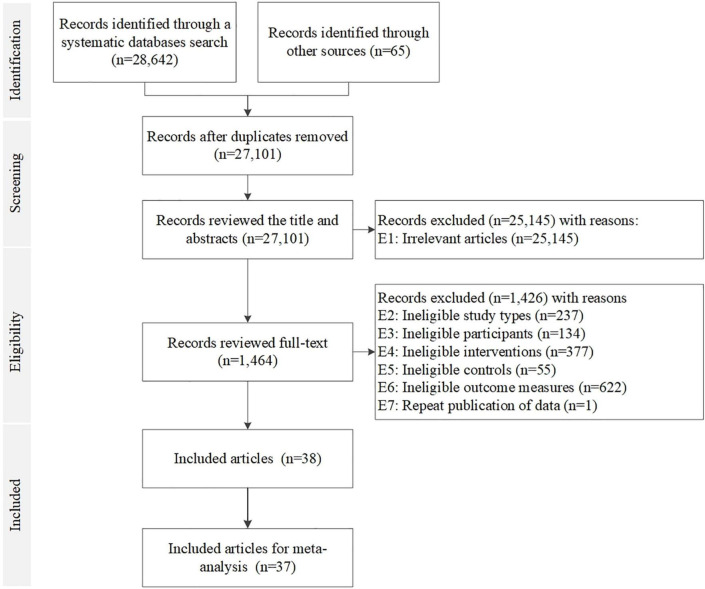
Flowchart of study selection procedures of RCTs included for meta-analysis.

A total of 28,642 records were identified after applying the keywords in the database search and 65 records were identified through other sources. Thirty eight records met the selection criteria of this review. All of them were included in the meta-analysis (17–53), except one study which did not report the units of any assessed outcomes (54). Figure 1 illustrates the selection process of included RCTs.

### Description of studies included in this review

All included RCTs were conducted in mainland China. Except for one paper published in English (51), all studies were published in Chinese. A total of 4,047 participants were recruited in the 38 included RCTs. One RCT (19) provided no information on the gender or age of the participants. four RCTs provided both sex and age information for all participants, but did not report data of each group (18, 25, 27, 54). Three trials investigated combined conditions: essential hypertension with carotid atherosclerosis (44), hypertension with hyperlipidemia (42) and hypertension with atherosclerosis (45). Eighteen trials recruited participants with various high blood pressure conditions, including hypertension (19, 20, 22, 34, 36, 38, 39, 41, 43, 50, 53), prehypertension (46), essential hypertension (30, 33, 35, 47), elderly patients with isolated systolic hypertension (28) and hypertension with dizziness (48); eight studies investigated atherosclerosis or cervical atherosclerosis (18, 23, 24, 26, 31, 40, 49, 52); six investigated coronary heart disease (17, 32, 37, 51, 54) and coronary heart disease with hyperlipemia (21); and three investigated coronary artery disease (25, 27, 29). The diagnostic criteria of all the involved CVD were provided in each paper accordingly (Table 1).

**TABLE 1:** Characteristics of included randomized controlled trials of Chinese herbal medicine on cardiovascular conditions.

Study ID	Setting	Condition	Diagnosis criteria of CVD	Sample size (T/C)	Gender (M/F)	Age	T vs. C	Trial duration	Inflammatory biomarkers
**CHM plus WM versus CHM placebo plus WM**									
Li et al. (28)	Hospital and clinic	EISH	JNC7 and the 2005 Chinese Hypertension Guidelines	135 (45/45/45); EoT 110 (37/35/38)	EoT T_1_: 14/23; T_2_: 12/23; C 13/25	EoT T_1_: 67.46 ± 6.35; T_2_: 66.60 ± 6.01; C: 67.29 ± 6.44	CHM + WM (T_1_) vs. CHM placebo + WM (C)	1–2 weeks run-in period + 4 weeks	hs-CRP
**CHM plus co-intervention versus the same co-intervention**									
Chen (17)	Hospital	CHD	Complied with the naming and diagnostic criteria for ischemic heart disease introduced by the International Cardiology and WHO	100 (50/50)	T: 20/30; C: 26/24	T: 53.89 ± 4.79; C: 56.89 ± 5.03	CHM + Routine vs. Routine	4 weeks	hs-CRP, IL-6
Chen and Cai (19)	Hospital (IP and OP)	H	Guidelines for the prevention and treatment of hypertension in China 2010	120 (60/60)	NA	NA	CHM + Routine vs. Routine	4 weeks	hs-CRP
Chen et al. (18)	University-affiliated teaching hospital	CAS	Color Doppler computer sonography confirmed (common carotid artery and internal carotid artery IMT > 1.0 mm, bifurcation IMT > 1.2 mm)	60 (30/30)	36/24	57 ± 4	CHM + Routine vs. Routine	16 weeks	hs-CRP
Dai (20)	Hospital	H	Guidelines for the prevention and treatment of hypertension in China 2010	160 (80/80)	T: 53/27; C: 55/25	T: 59.53 ± 6.32; C: 60.17 ± 5.28	CHM + WM + Lifestyle vs. WM + Lifestyle	8 weeks	IL-6, TNF-α
Ding and Hu (21)	University-affiliated teaching hospital	CHD + hyperlipidemia	Nomenclature and diagnostic criteria of ischemic heart disease; Chinese guidelines for prevention and treatment of dyslipidemia in adults 2007	56 (28/28)	T: 17/11; C: 15/13	T: 63.42 ± 10.27; 62.89 ± 10.16	CHM + WM + Lifestyle vs. WM + Lifestyle	12 weeks	hs-CRP, TNF-α, IL-1β, IL-18
Fan et al. (22)	University-affiliated teaching hospital	H	Diagnostic and grading criteria for hypertension of WHO/ISH 1999	60 (30/30)	T: 18/12; C: 20/10	T: 70.35 ± 8.42; C: 71.74 ± 7.65	CHM + WM vs. WM	16 weeks	hs-CRP, TNF-α
Huang et al. (24)	University-affiliated teaching hospital	CAS	Color Doppler ultrasonography confirmed cervical arteriovenous sclerosis (i.e., IMT ≥ 1.0 mm)	56 (29/27)	T: 17/12; C: 14/13	T: 46-75; C: 43–72	CHM + WM vs. WM	90 days	hs-CRP
Huang et al. (23)	University-affiliated teaching hospital (IP and OP)	CAS	Color Doppler ultrasonography confirmed cervical arteriovenous sclerosis (i.e., IMT ≥ 1.0 mm)	65 (34/31)	T: 19/15; C: 18/13	T: 43-72; C: 46–75	CHM + WM vs. WM	90 days	hs-CRP
Jia (25)	Hospital	Critical CAD	All patients met the clinical diagnostic criteria for critical coronary lesions after clinical CT examination.	89 (45/44)	46/43	67.8 ± 1.5	CHM + WM + Routine vs. WM + Routine	21 days (C)/30 days (T)	hs-CRP, IL-6
Jin et al. (26)	Hospital (IP and OP)	CAS	Color ultrasound examination confirmed carotid atherosclerosis (IMT ≥ 1.0 mm), intima media thickening (1.0 < IMT ≤ 1.2 mm), plaque formation (IMT ≥ 1.3 mm)	98 (50/48)	T: 26/24; C: 25/23	T: 57 ± 8.1; C: 58 ± 9.6	CHM + WM + Routine + Lifestyle vs. WM + Routine + Lifestyle	24 weeks	IL-6
Li and Long (27)	Hospital	Critical CAD	64-slice spiral CT confirmed critical coronary artery lesions (vascular diameter stenosis degree of 40% ∼ 70%)	156 (80/76)	82/74	48–79, average 54.8	CHM + WM + Routine vs. WM + Routine	1 month	hs-CRP, IL-6, TNF-α
Li et al. (30)	University-affiliated teaching hospital (IP and OP)	EH	Guidelines for the prevention and treatment of hypertension in China 2010	100(50/50)	T: 26/24; C: 28/22	T: 60.74 ± 11.79; C: 62.24 ± 10.24	CHM + Routine vs. Routine	4 weeks run-in period + 8 weeks	IL-6, TNF-α
Li et al. (29)	Hospital	CAS	Chinese guidelines for diagnosis and treatment of ischemic stroke 2010	136 (68/68)	T: 40/28; C: 38/30	T: 51.28 ± 2.91; C: 50.23 ± 2.88	CHM + WM vs. WM	3 months	hs-CRP
Liu (32)	Hospital	CHD	All the patients were examined by CT and met the diagnostic criteria for coronary artery disease with critical lesions	92(46/46)	T: 23/23; C: 21/25	T: 66.23 ± 1.14; C: 66.51 ± 1.09	CHM + WM + Routine + Lifestyle vs. WM + Routine + Lifestyle	30 days	hs-CRP, IL-6
Liu et al. (31)	Hospital	CAS	Chinese expert consensus on diagnosis and treatment of carotid atherosclerotic diseases in the elderly, and Guidelines for Ultrasound Examination of Blood Vessels and Superficial Organs	184 (92/92)	T: 50/42; C: 48/44	T: 65.21 ± 4.30; C: 63.19 ± 5.32	CHM + Routine vs. Routine	90 days	hs-CRP
Ma et al. (33)	University-affiliated teaching hospital	EH	Guidelines for the prevention and treatment of hypertension in China 2010	200 (100/100); Reported (30/30)	T: 58/42; C: 62/38	T: 54.24 ± 12.61; C: 54.14 ± 12.57	CHM + WM + Lifestyle vs. WM + Lifestyle	4 weeksk	IL-6, hs-CRP, MCP-1
Meng et al. (34)	Hospital (IP)	H	Guidelines for the prevention and treatment of hypertension in China 2010	200 (50/50/50/50)	A: 26/24; B: 24/26; C: 27/23; D: 25/25	A: 57.9 ± 2.0; B:58.1 ± 3.2; C: 57.8 ± 2.9; D: 57.4 ± 3.1	CHM + Routine (B) vs. Routine (A)	6 months	hs-CRP
							CHM + WM + Routine (D) vs. WM + Routine (C)		
Qian et al. (35)	Hospital	EH	Guidelines for the prevention and treatment of hypertension in China 2010	72 (36/36)	T: 18/18; C: 19/17	66.7 ± 8.6	CHM + WM vs. WM	2 weeks run-in period + 2 months	hs-CRP
Tian (36)	Hospital	H	ISH diagnostic criteria: 140 mmHg ≦ systolic pressure ≦ 160 mmHg 90 mmHg ≦ diastolic pressure;	84 (42/42)	T: 26/16; C: 24/18	T: 67.58 ± 9.42; C: 67.17 ± 8.87	CHM + WM + Lifestyle vs. WM + Lifestyle	4 weeks	IL-6, TNF-α
Wan and Li (37)	Hospital (IP)	CHD	Textbook of Internal Medicine	94 (47/47)	T:26/21; C: 27/20	T: 60.52 ± 5.44; C: 60.68 ± 5.37	CHM + WM vs. WM	4 weeks	hs-CRP
Wang et al. (38)	Hospital	H	Guidelines for the prevention and treatment of hypertension in China 2010	86 (42/44)	T: 26/16; C: 27/17	T: 54.19 ± 12.48; C: 53.48 ± 12.37	CHM + Routine vs. Routine	6 months	hs-CRP, TNF-α
Xie et al. (40)	Hospital (IP and OP)	CAS	The expert consensus of the 2003 radiology conference was used as the criterion: the thickness of carotid intima was >1.0 mm; Plaque was defined as: local IMT thickened and convex into the arterial lumen >0.5 mm, or increased by more than 50% compared with surrounding IMT, or IMT > 1.5 mm. Any of the above is considered as CAS.	135 (45/45/45)	T_1_: 24/21; T_2_: 26/19; C:25/20	T_1_: 63.92 ± 9.15; T_2_: 59.14 ± 9.90; C:61.32 ± 9.67	CHM + WM (T_1_) vs. WM	6 months	IL-10
Xie et al. (39)	Hospital	H	Guidelines for the prevention and treatment of hypertension in China 2005	120 (60/60)	T: 41/19; C: 38/22	T: 68.29 ± 4.62; C: 68.75 ± 6.01	CHM + Routine vs. Routine	8 weeks	IL-6, TNF-α
Xiong and Zhu (41)	Hospital (IP and OP)	H	Guidelines for the prevention and treatment of hypertension in China 2010	120 (40/40/40)	T_1_: 14/26; T_2_: 18/22; C: 20/20	T_1_: 68.30 ± 3.69; T_2_: 67.23 ± 5.06; C: 67.53 ± 3.68	CHM + WM vs. WM	3 months	hs-CRP
Xu et al. (42)	Hospital (IP and OP)	H + hyperlipidemia	Guidelines for the prevention and treatment of hypertension in China 2018; Chinese guidelines for prevention and treatment of dyslipidemia in adults 2016	80 (40/40)	T: 25/15; C: 19/21	T: 51.56 ± 10.87; C: 53.58 ± 10.47	CHM + WM vs. WM	12 weeks	IL-6, TNF-α
Yang and Huang (44)	University-affiliated teaching hospital	H + CAS	Guidelines for the prevention and treatment of hypertension in China 2010; ESC/EAS Guidelines for the management of dyslipidemias 2011	212 (106/106)	T: 56/50; C: 55/51	T: 55.83 ± 7.21; C: 55.37 ± 7.45	CHM + WM vs. WM	6 months	hs-CRP
Yang and Li (43)	Hospital (IP)	H	Guidelines for the prevention and treatment of hypertension in China 2005	90 (45/45)	T: 28/17; C: 29/16	T: 69.24 ± 3.87; C: 68.16 ± 3.69	CHM + Routine + Lifestyle vs. Routine + Lifestyle	32 days*	IL-6
Yao et al. (45)	Hospital	H + AS	Textbook of Internal Medicine	108 (54/54)	T: 22/32; C: 24/30	T: 61.08 ± 8.13; C: 60.75 ± 7.24	CHM + WM + Lifestyle vs. WM + Lifestyle	6 months	IL-6, TNF-α
Yu (54)	Hospital	CHD	ISFC/WHO diagnostic criteria for CHD 1979	60 (30/30)	41/19	51.5 ± 9.3	CHM + Routine vs. Routine	10 weeks	IL-6, TNF-α
Zeng et al. (46)	Hospital and clinic	PHT	JNC7 diagnostic criteria for PHT	120 (40/40/40)	T: 25/15; C_1_: 16/24: C_2_: 21/19	T: 39.13 ± 6.66; C_1_: 40.90 ± 6.60: C_2_: 42.40 ± 7.08	CHM + Lifestyle (T) vs. Lifestyle (C_2_)	0.5 year	hs-CRP
Zhang et al. (47)	Hospital (IP and OP)	EH	Guidelines for the prevention and treatment of hypertension 2005	120 (70/50); EoT 119 (70/49)	T: 41/29; C: 28/22	T: 51.2 ± 6.1; C: 50.3 ± 7.5)	CHM + WM vs. WM	3 months	hs-CRP
Zhang et al. (48)	University-affiliated teaching hospital	H + dizziness	Guidelines for the prevention and treatment of hypertension 2010	138 (69/69); EoT 130 (63/67)	T: 19/44; C: 30/37	T: 65.84 ± 10.47; C: 63.70 ± 10.40	CHM + Routine vs. Routine	28 days	hs-CRP, IL-6, sICAM-1, sVCAM-1
Zheng et al. (49)	Hospital	AS	1979 WHO diagnostic criteria; coronary artery stenosis ≥ 30% or other vascular stenosis ≥ 50%	30 (10/10/10); EoT 27 (9/9/9)	T_1_: 6/4; T2: 7/3; C: 7/3	T_1_: 63.1 ± 9.5; T_2_: 60.1 ± 12.4; C: 61.2 ± 11.5	CHM + Routine (T_1_: Huo Xue) vs. Routine	1 month	hs-CRP, TNF-α
							CHM + Routine (T_2_: Huo Xue Jie Du) vs. Routine		
Zhou (50)	Hospital	H	Guidelines for the prevention and treatment of hypertension 2010	90 (30/30/30)	T_1_: 18/12; T_2_: 16/14; C: 18/12	T_1_: 51.34 ± 5.38; T_2_: 50.83 ± 5.14; C: 51.67 ± 5.61	CHM + WM (T_1_) vs. WM	6 months	hs-CRP, IL-8
Zhu et al. (51)	University-affiliated teaching hospital (IP)	CHD	Diagnostic criteria for CHD followed the naming and diagnostic criteria of ischemic heart disease issued by WHO	101 (45/56)	T: 20/25; C: 24/32	T: 71 ± 8; C: 70 ± 9	CHM + Routine vs. Routine	2 weeks	hs-CRP, IL-6, IL-10
Zhu et al. (52)	University-affiliated teaching hospital (IP and OP)	AS	Ultrasonic Medicine (textbook), 6th Edition	60 (30/30)	T: 17/13; C: 16/14	T: 65.33 ± 8.06; C: 63.3 ± 7.83	CHM + Routine vs. Routine	8 weeks	IL-6, TNF-α
Zuo et al. (53)	University-affiliated teaching hospital (IP and OP)	H	Guidelines for the prevention and treatment of hypertension 2010	60 (30/30)	T: 15/15; C: 16/14	T: 60.67 ± 10.89; C: 62.00 ± 11.34	CHM + Routine vs. Routine	28 days	TNF-α, IL-6

ApoB/A, apolipoprotein B/A; C, control group; CAD, coronary artery disease; CAS, carotid atherosclerosis; CH, Chinese; CHD, coronary heart disease; CHM, Chinese herbal medicine; hs-CRP, hyper sensitivity C-reactive protein; CVD, cardiovascular diseases; EH, essential hypertension; EISH, elderly patients with isolated systolic hypertension; EAS, the European Atherosclerosis Society; EN, English; EoT, end of treatment; ESC, the European Society of Cardiology; F, female; H, hypertension; IL, interleukin; IMT, intima – media thickness; ISFC, International Society and Federation of Cardiology; ISH, International Society of Hypertension Global; IP, inpatient; JNC7, the Seventh Report of the Joint National Committee; JNC8, the eighth Report of the Joint National Committee; M, male; MCP-1, monocyte chemotactic protein-1; OP, outpatient; PHT, prehypertension; sICAM-1, soluble intercellular adhesion molecule-1; sVCAM-1, soluble circulating vascular adhesion molecule-1; T, treatment group; TNF, tumor necrosis factor; WHO, the World Health Organization; WM, western medicine;. *The study reported contradictory descriptions of the duration of intervention administration: “continuous treatment for 8 weeks as one course” and “continuous treatment for 4 courses (32 days).” While this may have been a typographical error, attempts to contact the authors to clarify this were not possible as email or telephone details were not provided in the paper.

Most of the included RCTs were composed of two arms, however, two studies containing more than two arms and making two comparisons were also included (34, 49) bringing the total number of comparisons in this review to 40. In these 38 studies, one study (34) formed two comparisons with different co-interventions; one compared CHM plus routine treatment with routine treatment alone, while the other compared CHM plus WM plus routine treatment versus WM plus routine treatment; one study (49) formed two comparisons with different CHM interventions: i.e., either Huo Xue or Huo Xue Jie Du plus routine treatment compared with routine treatment alone; the other co-interventions involved in the remaining 35 studies were WM (22–24, 29, 35, 37, 40–42, 44, 47, 50), routine treatment (17–19, 30, 31, 38, 39, 48, 51–54), lifestyle changes (46), WM + routine treatment (25, 27), WM + lifestyle changes (20, 21, 33, 36, 45), routine treatment + lifestyle changes (43), WM + routine treatment + lifestyle changes (26, 32). Despite the duration of treatment and control intervention being different in one trial (25) (21 days for the control and 30 days for the treatment), the treatment duration of all the groups in the remaining studies were the same and varied between 4 weeks to 6 months and no study reported follow-up after treatment. The characteristics of included RCTs are summarized in Table 1 and details of interventions are provided in Supplementary Table 2.

### Risk of bias assessment

Greater than or equal to 50% of included studies had low risk of bias in the domains of random sequence generation, detection, attrition, reporting and other bias and 97% had a high risk of performance bias.

#### Selection bias

Randomization of participants in all of the included studies were stated to be performed *via* random sequence generation. However, two studies performed quasi-random (alternation) (19, 43) (high risk), 17 studies fail to specify the randomization method (17, 18, 23, 24, 27, 29–32, 34, 35, 39, 40, 42, 44, 53, 54) (unclear risk); and the rest (19 studies) provided appropriate randomization methods (e.g., random number table method) (20–22, 25, 26, 28, 33, 36–38, 41, 45–52). None of the included studies provided adequate information on how randomized numbers were allocated to participants and achieved an unclear risk of bias on the allocation concealment.

#### Performance bias

One study (28) used a placebo to achieve blinding of participants and personnel (low risk), whereas the remaining studies, irrespective of whether they stated the study to be blinded or not, could not achieve blinding due to different forms of interventions used in two groups (e.g., decoction versus tablet, capsule versus tablet) (high risk).

#### Detection bias

None of the included RCTs provided sufficient information on the blinding of outcome assessors. However, as all the included outcome measures in this review are laboratory results, the outcome measurements were not likely to be influenced by lack of blinding. Thus, they have low risk of detection bias.

#### Attrition bias

High attrition bias was noticed in two studies: one study (31) performed per-protocol analysis of treatment intervention and stated that it excluded participants who experienced severe adverse events; the reported participant number (69/69) given in the Methods section of one study (48) was not equal to that presented in the Results section (63/67) which indicated that outcome data were missing in both intervention groups (6 versus 2, 8.69% versus 2.89%), but reasons were not clearly reported to be able to assess if they were balanced across groups. Additionally, unclear risk of bias was noticed in 17 studies due to poor statements describing drop out numbers and blurred descriptions of whether they performed pre-protocol analysis methods (21, 25, 26, 32, 34, 36–40, 44–46, 49, 51–53).

#### Reporting bias

Trial protocol was not identified in any of the included studies. After comparing the described outcomes in the “Materials and methods” section to those reported in the “Results” section, five studies failed to report findings for all the outcome measures described in “Materials and methods” section (35, 40, 46, 47, 54) (high risk); five studies reported all findings described in the “Materials and methods” section but added other outcomes in Results section (17, 19, 28, 49, 51) (unclear risk); and the rest of the RCTs provide consistent information in those two sections (low risk).

#### Other bias

“Other bias” was assessed from two aspects: authors’ description of relevant clinical and demographic baseline parameters and relevant funding information. The authors from one study (18) did not provide a clear statement of baseline values (unclear risk); while the remaining studies claimed to have matched control and treatment groups at baseline: 31 studies stated the *p*-value > 0.05 for assessed parameters (17, 19–26, 28–34, 36–46, 49, 50, 52, 54) and two studies reported statistical baseline comparison data in tables (35, 53) and four did not report relevant statistical data (27, 47, 48, 51). Twelve studies did not provide sufficient information on funding (17, 23, 25, 27, 31, 32, 36, 39, 43, 47, 53, 54) (unclear risk) and the rest were funded by government programs or organizations.

**FIGURE 2:**
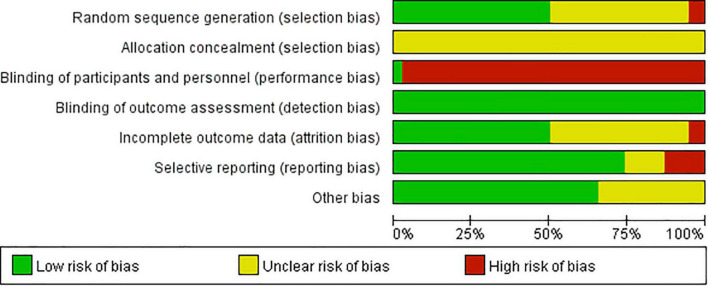
Risk of bias graph: review authors’ judgments about each risk of bias item presented as percentages across all included studies.

**FIGURE 3:**
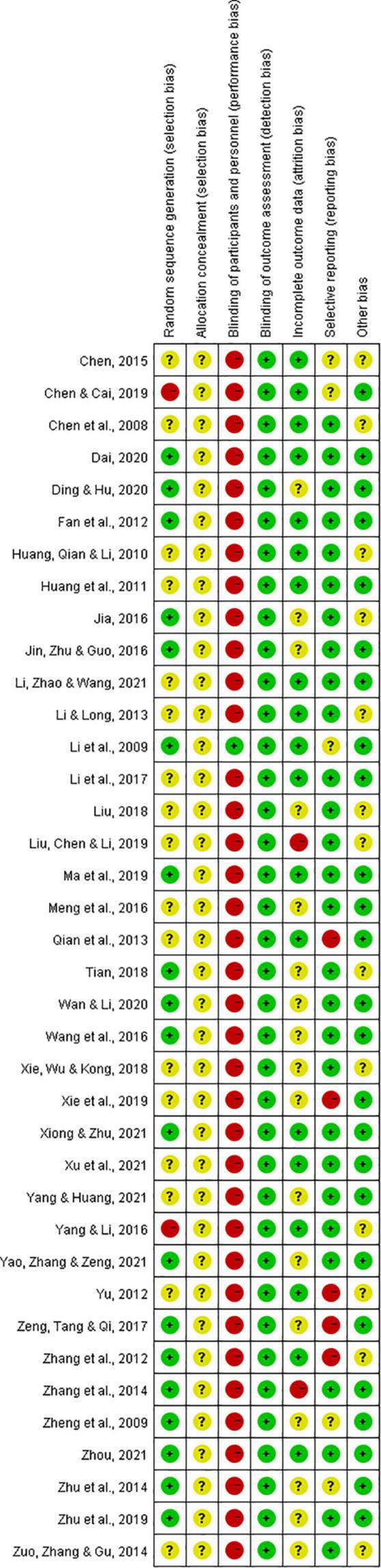
Risk of bias summary: review authors’ judgments about each risk of bias item for each included study.

The risk of bias results are summarized and presented in Figures 2, 3.

### Meta-analysis results

None of the included studies compared CHM with no treatment. One study (28) employed a placebo control and compared CHM plus WM with CHM placebo plus WM, whereas all of the other included studies compared CHM plus co-intervention to the same co-intervention to investigate the additive effects of CHM on the intervention. Two outcomes from four studies (32, 40, 49, 51) had improperly matched baseline values of the parameters measured and did not address this problem in data analyses (i.e., they are not reporting changes from baseline between control and treatment arms) (Supplementary Table 3), thus they were not included in the meta-analysis. All the included data were categorized by interventions and sub-grouped by outcome measures.

#### Chinese herbal medicine plus western medicine versus Chinese herbal medicine placebo plus western medicine

##### Effects on high sensitivity c-reactive protein

One study (28) compared Jiang ya capsule plus WM with CHM placebo plus WM and reported no significant differences in hs-CRP levels after a 4-week intervention period (MD 0.04, 95% CI −1.66 to 1.74).

#### Chinese herbal medicine plus co-intervention versus the same co-intervention

##### Summary of effects

Meta-analysis results indicated significant additive effects of CHM on reducing hs-CRP (SMD −2.05, 95% CI −2.55 to −1.54, *I*^2^ = 96%, *p* < 0.00001) (17–19, 21–25, 27, 29, 31–35, 37, 38, 41, 43, 44, 46–50), IL-6 (SMD −1.14, 95% CI −1.63 to −0.66, *I*^2^ = 95%, *p* < 0.00001) (17, 20, 25–27, 30, 33, 36, 42, 43, 45, 48, 51–53) and TNF-α levels (SMD −0.88, 95% CI −1.35 to −0.41, *I*^2^ = 92%, *p* = 0.0002) (20–22, 27, 30, 36, 38, 39, 42, 45, 49, 52, 53) when combined with other interventions (Supplementary Figures 1–3). Table 2 summarizes the meta-analysis results for hs-CRP, IL-6 and TNF-α in CHM plus co-intervention groups (before and after treatment), in co-intervention groups (before and after treatment), and between two groups (at the baseline and at the end of treatment). Detailed statistical analysis data of Table 2 are provided in Supplementary Figures 4–12. Sensitivity analysis indicated that no single study significantly altered the direction or magnitude of the pooled estimates.

**TABLE 2:** Hs-CRP, IL-6, and TNF-α in Chinese herbal medicine plus co-intervention groups and the same co-intervention groups.

Inflammatory biomarkers	Between two groups at the baseline			T (before and after treatment)			C (before and after treatment)			Between two groups at the end of treatment		
				
	No. of comparisons (sample size)	Total (95% CI)	*I*^2^, *p*	No. of comparisons (sample size)	Total (95% CI)	*I*^2^, *p*	No. of comparisons (sample size)	Total (95% CI)	*I*^2^, *p*	No. of comparisons (sample size, T/C)	Total (95% CI)	*I*^2^, *p*
**hs-CRP**	24 (2285)	SMD −0.00 [−0.08, 0.08]	*I*^2^ = 0%, *p* = 0.98 (Supplementary Figure 4)	24 (1160)	SMD −4.38 [−5.23, −3.53]	*I*^2^ = 98%, *p* < 0.00001 (Supplementary Figure 5)	24 (1125)	SMD −2.45 [−3.04, −1.86]	*I*^2^ = 97%, *p* < 0.00001 (Supplementary Figure 6)	26 (1250/1215)	SMD −2.05 [−2.55, −1.54]	*I*^2^ = 96%, *p* < 0.00001 (Supplementary Figure 1)
**IL-6**	14 (1376)	SMD 0.01 [−0.10, 0.11]	*I*^2^ = 0%, *p* = 0.88 (Supplementary Figure 7)	14(684)	SMD −2.55 [−3.43, −1.67]	*I*^2^ = 98%, *p* < 0.00001 (Supplementary Figure 8)	14 (692)	SMD −1.46 [−2.12, −0.79]	*I*^2^ = 97%, *p* < 0.0001 (Supplementary Figure 9)	15 (734/742)	SMD −1.14 [−1.63, −0.66]	*I*^2^ = 95%, *p* < 0.00001 (Supplementary Figure 2)
**TNF-α**	14 (1166)	SMD 0.04 [−0.08, 0.15]	*I*^2^ = 0%, *p* = 0.52 (Supplementary Figure 10)	14 (584)	SMD −2.03 [−2.88, −1.77]	*I*^2^ = 97%, *p* < 0.00001 (Supplementary Figure 11)	14 (582)	SMD −1.13 [−1.78, −0.49]	*I*^2^ = 96%, *p* = 0.0006 (Supplementary Figure 12)	14 (584/582)	SMD −0.88 [−1.35, −0.41]	*I*^2^ = 92%, *p* = 0.0002 (Supplementary Figure 3)

C, control group; CI, confidence intervals; hs-CRP, high sensitivity c-reactive protein; I^2^, test for heterogeneity in percentage; IL-6, interleukin-6; MD, mean difference; SMD, standardized mean difference; T, treatment group; TNF-α, tumor necrosis factor-α. Three studies reported hs-CRP data as ng/L [Ma et al., (33); Wang et al., (38); Yang and Li, (43)] and the rest reported data as mg/L. One study [Xu et al., (42)] reported IL-6 as mg/L, and the rest reported data as pg/mL or ng/L. Two studies reported TNF-α data as mmol/L [Tian, (36); Zuo et al., (53)], four studies reported TNF-α levels as pg/mL or ng/L [Fan et al., (22); Li and Long, (27); Wang et al., (38); Xie et al., (39)], two studies as ng/mL [Li et al., (30); Zhu et al., (52)], and one as mg/L [Zheng et al., (49)]. Two studies [Chen, (17); Zeng et al., (46)] did not provide baseline data. Detailed statistical analysis of data in this Table are provided in Supplementary Figure 4–12.

Seven other inflammatory biomarkers were assessed in five separate studies.

One study (33) compared levels of MCP-1 following treatment with CHM (Ban Xia Bai Zhu Tian Ma Tang) plus co-intervention (WM + lifestyle) versus co-intervention alone. After 4-weeks intervention, MCP-1 levels in the treatment group were significantly lower than that in the control group (MD −31.32, 95% CI −55.15 to −7.49).

One study (21) assessed levels of IL-1β and IL-18 with a 12-weeks intervention period and results showed significant reduction of IL-1β (MD −0.17, 95% CI −0.24 to −0.10) and IL-18 levels (MD −27.89, 95% CI −43.04 to −12.74) in the combined group (Shu Gan Qing Zhi Fang + WM + lifestyle changes) when compared to the control group (WM + lifestyle).

One study (48) assessed sICAM-1 and sVCAM-1 (markers of endothelial activation) by comparing CHM (Shu Nao Xin Di Wan) plus routine treatment to routine treatment. The results showed that CHM had a significant additive effect of reducing both soluble ICAM-1 levels (MD −40.03, 95% CI −76.72 to −3.34) and soluble VCAM-1 levels (MD −0.55, 95% CI −1.04 to −0.06) after a 28-day intervention.

One study (50) assessed IL-8 levels by comparing CHM (Tong Mai Jie Du Fang) plus WM (nifedipine extended-release tablets and atorvastatin calcium tablets) to WM and after 6-month intervention IL-8 level reduced significantly (MD −6.36, 95% CI −7.78 to −4.94) in the co-treatment group.

Two studies assessed IL-10 levels, reporting different results: one study (40) reported an increase in IL-10 levels with an increase significantly higher in the CHM (Shen Qi Mai Xin Tong Capsule) plus WM group compared to WM alone (MD 14.37, 95% CI 5.01 to 23.73); whereas the other study (51) reported a reduction in IL-10 levels where the reduction was greater for the control group (routine treatment) compared to the co-treatment group (Lian Dou Qing Mai Recipe plus routine treatment) (MD 0.17, 95% CI 0.10 to 0.24).

##### Subgroup analysis

Similar to the meta-analysis, subgroup analyses of covariates (co-intervention, condition and trial duration) identified significant differences. Subgroup analysis of co-interventions showed that CHM significantly reduced hs-CRP when comparing different co-interventions, conditions and trial duration, and IL-6 and TNF-α when comparing different trial duration (Table 3). Detailed statistical subgroup analysis of hs-CRP, IL-6 and TNF-α based on different co-interventions is presented in Supplementary Figures 13–15, based on different conditions in Supplementary Figures 16–18, and based on different trial duration in Supplementary Figures 19–21.

**TABLE 3:** Subgroup analysis of hs-CRP, IL-6, and TNF-α of Chinese herbal medicine plus co-intervention group and the same co-intervention groups.

Subgroups		hs-CRP		IL-6		TNF-α	
				
		Subtotal (95%, CI)	*I* ^2^	Subtotal (95%, CI)	*I* ^2^	Subtotal (95%, CI)	*I* ^2^
Co-interventions	WM	SMD −2.74 (−3.78, −1.70)*	97% (22–24, 29, 35, 37, 41, 44, 47, 50)	MD −0.37 (−1.68, 0.94)	NA (42)	SMD −1.02 (−2.12, 0.07)	89% (22, 42)
	Lifestyle	MD −0.50 (−0.88, −0.12)*	NA (46)	NA	NA	NA	NA
	Routine	SMD −1.32 (−2.10, −0.55)*	96% (17–19, 31, 34, 38, 48, 49)	SMD −0.61 (−0.91, −0.31)*	66% (17, 30, 48, 51–53)	SMD −0.42 (−0.68, −0.16)*	43% (30, 38, 39, 49, 52, 53)
	WM + Lifestyle	SMD −1.23 (−1.63, −0.83)*	0% (21, 33)	SMD −0.96 (−1.29, −0.62)*	60% (20, 33, 36, 45)	SMD −1.10 (−1.73, −0.47)*	88% (20, 21, 36, 45)
	Routine + Lifestyle	MD −0.65 (−0.97, −0.33)*	NA (43)	MD −0.41 (−0.66, −0.16)*	NA (43)	NA	NA
	WM + Routine	SMD −2.83 (−3.57, −2.10)*	82% (25, 27, 34)	SMD −4.28 (−4.74, −3.82)*	0% (25, 27)	MD −21.06 (−22.93, −19.19)*	NA (27)
	WM + Routine + Lifestyle	MD −3.80 (−4.25, −3.35)*	NA (32)	MD −3.12 (−4.69, −1.55)*	NA (26)	NA	NA
Conditions	High blood pressure	SMD −2.14 (−2.88, −1.40)*	96% (19, 22, 33–35, 38, 41, 46–48, 50)	SMD −0.64 (−0.93, −0.35)*	74% (20, 30, 33, 36, 42, 43, 48, 53)	SMD −0.70 (−0.99, −0.41)*	73% (20, 22, 30, 36, 38, 39, 42, 53)
	Coronary artery disease	SMD −2.44 (−2.77, −2.10)*	0% (25, 27)	SMD −4.28 (−4.74, −3.82)*	0% (25, 27)	MD −21.06 (−22.93, −19.19)*	NA (27)
	Atherosclerosis	SMD −1.05 (−2.01, −0.09)*	95% (18, 23, 24, 31, 49)	SMD −0.69 (−1.01, −0.37)*	0% (26, 52)	SMD −0.10 (−0.81, 0.62)	60% (49, 52)
	Coronary heart disease	SMD −3.00 (−5.11, −0.88)*	98% (17, 21, 32, 37)	SMD −0.87 (−1.79, 0.05)	90% (17, 51)	MD −0.72 (−1.23, −0.21)*	NA (21)
	Hypertension + coronary heart disease	MD −3.39 (−3.74, −3.04)*	NA (44)	NA	NA	NA	NA
	Hypertension + atherosclerosis	NA	NA	MD −8.29 (−11.84, −4.74)*	NA (45)	MD −17.13 (−20.30, −13.96)*	NA (45)
Trial duration	<12 weeks	SMD−1.99 (−2.87, −1.11)*	97% (17, 19, 25, 27, 32, 33, 35, 37, 43, 48, 49)	SMD −1.29 (−1.89, −0.68)*	95% (17, 20, 25, 27, 30, 33, 36, 43, 48, 51–53)	SMD −0.73 (−1.40, −0.06)*	94% (20, 27, 30, 36, 39, 49, 52, 53)
	≥12 weeks	SMD−2.09 (−2.73, −1.45)*	96% (18, 21–24, 29, 31, 34, 38, 41, 44, 46, 47, 50)	SMD −0.60 (−1.06, −0.15)*	72% (26, 42, 45)	SMD −1.12 (−1.71, −0.53)*	86% (21, 22, 38, 42, 45)

CI, confidence intervals; I^2^, test for heterogeneity in percentage; MD, mean difference; SMD, standardized mean difference; *, significant difference (p < 0.05). Subgroup analysis of hs-CRP, IL-6 and TNF-α based on different co-interventions are presented in Supplementary Figures 13–15, that based on different conditions in Supplementary Figures 16–18, and based on different trial duration in Supplementary Figures 19–21.

### Safety

One study (29) compared CHM (Hua Ban Fang) plus WM (atorvastatin calcium tablets and aspirin enteric sol sac) after a 3-month intervention, and the co-treatment group showed a significantly lower adverse event rate than the WM group (χ^2^ = 8.84, *p* < 0.01). Sixteen of the included studies assessed safety and reported no significant difference between the groups, with no vital adverse events in the treatment group (18, 19, 22, 26, 28, 30, 33, 42–47, 49, 50, 52). Details are listed in Supplementary Table 4.

### Publication bias

Visual inspection of funnel plots revealed potential asymmetry for hs-CRP, IL-6 and TNF-α (Supplementary Figure 22), however, after further quantifying the funnel plots using Egger’s and Begg’s tests, a significant influence from small studies occurred in hs-CRP (Egger’s: *p* = 0.001, Begg’s: *p* = 0.001) and IL-6 (Egger’s: *p* = 0.020, Begg’s: *p* = 0.255), but not in TNF-α (Egger’s: *p* = 0.761, Begg’s: *p* = 0.784).

### Frequently used herbs

Among the 37 studies included for meta-analyses, 35 studies reported lowering effects of CHM for at least one inflammatory biomarker. One study employed a single herb (Da huang, Rhei Radix et Rhizoma) as CHM intervention (54). The ingredients of formulae were not provided in four publications (34, 44, 47, 48) and could not be identified from Zhong Yi Fang Ji Da Ci Dian (14). The most frequently used herbs present within the remaining 32 formulas were identified and the top 3 were Dan shen (Salviae Miltiorrhizae Radix et Rhizoma, *n* = 16), Huang qi (Astragali Radix, *n* = 12) and Gan cao (Glycyrrhizae Radix et Rhizoma, *n* = 10), followed by Dang gui (Angelicae Sinensis Radix, *n* = 9), Chuan xiong (Chuanxiong Rhizoma, *n* = 8), Bai zhu (Atractylodis Macrocephalae Rhizoma, *n* = 7), Niu xi (Achyranthis Bidentatae Radix, *n* = 7) and Tian ma (Gastrodiae Rhizoma, *n* = 7).

## Discussion

To the best of our knowledge, this is the first meta-analysis that has sought to provide an assessment of the clinical effects of CHM, alone and in combination with other treatments, on inflammatory biomarkers in individuals with underlying CVD. Findings from 38 included studies (totaling 4,047 participants) revealed that CHM may significantly decrease plasma levels of inflammatory biomarkers relevant to CVD risk (hs-CRP, IL-6, and TNF-α) as well as the monocyte chemokine MCP-1, and markers of vascular endothelial cell activation, sICAM-1 and sVCAM-1. In contrast, the analysis did not reveal any clinical effects of CHM in combination with a standard intervention on the levels of the anti-inflammatory cytokine IL-10, based on data from two included studies. Categorical subgroup analysis identified substantial between-group differences for covariates, suggesting that the hs-CRP attenuating effect did not alter among different covariates (co-interventions, conditions, and trial durations), whereas the anti-inflammatory effects related to IL-6 and TNF-α may vary depending on co-interventions and conditions. Most included formulae for meta-analyses were identified to have lowering effects on at least one inflammatory biomarker, except those from two studies (40, 49). Dan shen (Salviae Miltiorrhizae Radix et Rhizoma), Huang qi (Astragali Radix), and Gan cao (Glycyrrhizae Radix et Rhizoma) were determined to be the most frequently used herbs in the included formulae. The safety analysis indicated that CHM formulae were safe to use as an add-on therapy to WM, routine treatment and lifestyle changes in CVD patients.

Cardiovascular disease is a leading cause of death in PWH (55) and rates of CVD appear to be increased in PWH compared to age-matched, HIV-uninfected individuals (56). Traditional assessments for CVD risk, including lipid profiles (i.e., total cholesterol and high-density lipoprotein cholesterol), present in a comparable way irrespective of HIV status (55). Together with the observation that increased CVD risk in PWH is independent of traditional risk factors [reviewed in (5)], they may not be the most appropriate primary outcomes to investigate mechanisms of altered risks of CVD in PWH. In contrast, generalized inflammation biomarkers are predictive of CVD events in PWH (57). The characteristics of untreated HIV infection include increases in the levels of inflammatory biomarkers (e.g., IL-6 and hs-CRP) and adhesion molecule expression relevant to atherosclerotic plaque formation. IL-6 and hs-CRP were also strongly associated with the development of AIDS events and opportunistic diseases (57, 58). These factors were also determined to be important in the CVD pathogenesis. Inflammatory biomarkers and thrombosis have the potential to improve CVD risk stratification beyond traditional and HIV-specific factors (59). Additionally, low physical functional status in PWH was associated with significantly higher IL-6 and TNF-α levels (56). Thus, we considered that inflammatory biomarkers are suitable to evaluate CHM as an adjunctive CVD prevention strategy for individuals with HIV infection and may be useful to explain the potential effects of CHM on improvement of quality of life in future studies.

Inflammation plays a crucial role in the pathophysiology of CVD, yet it is not known whether CHM can improve inflammatory outcomes and markers of innate immune activation relevant to CVD. We initially intended to evaluate this in PWH, since there is considerable evidence that residual inflammation and innate immune activation contributes to CVD in these individuals independently of traditional risk factors such as hypercholesterolemia. However, preliminary surveys revealed that there was an absence of studies on the anti-inflammatory effects of CHM in PWH to enable a systematic review or meta-analyses to be performed to address this question. Most current meta-analyses either focused on CHM for the treatment of AIDS (60) or investigated the effects of CHM with or without co-interventions on CVD management, but their favorable outcomes were blood lipid profiles (61), quality of life (62) or other specific parameters of the investigated conditions (e.g., blood pressure) (63), and not inflammatory biomarkers. The available studies were not sufficient to conduct a systematic review: only two RCTs and one parallel controlled study were returned after a comprehensive search of English and Chinese databases. Even though the number of relevant studies were limited, the results were suggestive of potential therapeutic effects. Wu et al. (64) lead a university-affiliated-hospital-based RCT conducted in Fujian China. Seventy individuals with AIDS with lung infection were treated with either WM alone (Cephalosporin antibiotics + Levofloxacin + Fluconazole) or in combination with CHM (Ai Fei Yi Hao). After 14-days of intervention, results indicated a significant decrease in hs-CRP in the co-treatment group (MD −8.64, 95% CI −15.06 to −2.22). Hs-CRP was also assessed by a hospital-based parallel controlled study (65) conducted in Zhengzhou, China, in which 102 individuals with AIDS with lung infection were allocated to a routine treatment group (anti-viral drug regimen and anti-microbial therapies) and a CHM (Fu Zheng Qing Fei Tang) plus routine treatment group. A significant additive effect of CHM on lowering hs-CRP was reported compared to the routine treatment group (MD −1.17, 95% CI −1.91 to −0.43), but this paper failed to report the intervention period. These results are consistent with the findings of the present meta-analysis of CHM intervention’s additive effects on hs-CRP levels in HIV-uninfected individuals despite the differences in co-intervention regimes, conditions and trial durations. Additionally, one study (66) evaluated the effects of CHM on IL-6 levels with reference to placebo control (*n* = 70) by a university-affiliated-hospital-based RCT conducted in Guangxi, China. Two formulas were provided to participants in the CHM group (*n* = 140) based on their Chinese medicine differential diagnosis: Shen Ling Fu Zheng Capsules for “Qi and Blood deficiency syndrome,” Qing Du Capsules for “damp-heat syndrome.” CHM significantly reduced serum IL-6 levels compared to placebo after an 18-month intervention (MD −2.62, 95% CI −4.86 to −0.38). Similar results were seen in our meta-regression of IL-6 levels. Our finding also bring attention to the possible categorical difference triggered by co-intervention regimes and types of CVD. The present systematic review is, to our knowledge, the first meta-analysis that sought to provide an assessment on clinical effects of CHM (with or without co-interventions) on inflammatory biomarkers among cardiovascular condition populations.

There is comparatively little data on whether reduction of inflammatory biomarkers following therapeutic interventions correlates with improved outcomes with respect to risk and progression of CVD, especially in PWH. However, given the association between higher levels of inflammatory biomarkers and the risk of CVD it is reasonable to assume that treatments that reduce inflammation will be beneficial in this regard. However, it is important that direct evidence for this is obtained by examining how reduction in inflammation impacts more robust measures of coronary artery disease, including surrogate measures of atherosclerosis such as carotid artery intima-media thickness and pulse wave velocity, direct measures of atherosclerotic disease such as imaging of plaques, and cardiovascular events.

Among the 32 formulas with clear identified ingredients, Dan shen (Salviae Miltiorrhizae Radix et Rhizoma), Huang qi (Astragali Radix) and Gan cao (Glycyrrhizae Radix et Rhizoma) were the most frequently used herbs in the included formulas with anti-inflammatory effects. Dan shen is the root of *Salvia miltiorrhiza* Bge. (10). The extract of Dan shen and its main constituents (i.e., salvianolic acid B, tanshinone IIA and protocatechuic acid) showed attenuating effects on the expression of VCAM-1 and ICAM-1, inhibition on the release of sVCAM-1 and sICAM-1 in human umbilical vein endothelial cells, and strong downregulating properties on TNF-α induced expression of CD40 (67). Huang qi, is derived from the root of *Astragalus membranaceus* (Fisch.) Bge. var. *mongholicus* (Bge.) Hsiao and *Astragalus membranaceus* (Fisch.) Bge. (10). One of its major bioactive constituents, astragalosides have been reported to induce T cell activation, regulate T cell balance, enhance CD45 phosphatase activity, inhibit pro-inflammatory cytokines and the NFκB pathway (68). Gan cao, also known as licorice, originates from *Glycyrrhiza uralensis* Fisch., *Glycyrrhiza inflata* Bat. and *Glycyrrhiza glabra* L. (10). Both the bioactive compounds present in, and metabolites of, Gan cao have shown anti-inflammatory properties: glycyrrhizin, a bioactive compound, interacts with the lipid bilayer of RAW 264.7 cells to attenuate inflammatory responses in macrophages (69); β-glycyrrhetinic acid, the major metabolite of glycyrrhizin (70), can reduce inflammatory responses by inhibiting glucocorticoid metabolism (71). In the context of PWH, some constituents from Dan shen and Gan cao have been reported to have effects *in vitro* on HIV infection. Lithospermic acid and lithospermic acid B are two selective HIV-1 integrase inhibitors present in Dan shen which strongly suppressed the acute HIV-1 infection of H9 cells (72). Lithospermic acid is also a non-covalent competitive inhibitor of nucleic acid binding to HIV nucleocapsid making it a potential representative of a new class of HIV inhibitor (73). Gan cao was identified to be one of the most frequent clinically used herbs in PWH with hyperlipidemia (31.58%) in a population-based study conducted in Taiwan (11). Glycyrrhizic acid from Gan cao inhibits the replication of HIV (74); glycocoumarin and lico-pyranocoumarin inhibit giant cell formation in HIV-infected cell cultures (71, 75). These two herbs may be worth exploring further to discover more options on driving CVD in PWH of dyslipidemia.

*I*^2^ is a measure of the extent of heterogeneity (12). The *I*^2^ of the three main outcomes were 96% (hs-CPR), 95% (IL-6) and 92% (TNF-α), which indicated considerable heterogeneity. Subgroup analysis was applied to further explore heterogeneity. After subgroup analysis of the included studies based on the co-interventions and conditions, several had an *I*^2^ below 50% (indicating less than substantial heterogeneity): TNF-α of the routine treatment subgroup (43%), hs-CRP of the WM plus lifestyle subgroup (0%), IL-6 of the WM plus routine treatment subgroup (0%), hs-CRP and IL-6 of the coronary artery disease subgroup (0 and 0%), IL-6 of the atherosclerosis subgroup (0%). Subgroup analysis of trial duration did not make a noticeable difference to *I*^2^. The statistical heterogeneity showed variation in intervention effects in these included studies. This review aimed to take a broader perspective in the intervention effects of CHM on inflammatory biomarkers in CVD. Therefore, clinical variation may possibly lead to the high heterogeneity: (1) CVD included diverse diseases. Due to the limited number of included studies, the categorization of condition subgroup analysis was not very specific and the inflammatory drivers of these diseases may be different; (2) similar to that observed with condition, even though subgroup analysis of the co-interventions reduced the *I*^2^ to a certain level, the diversity of the control groups could contribute to the high heterogeneity; (3) distinctiveness of the CHM interventions should also be considered. The herbal ingredients of each formula are different. These adhesion interventions may not have the same intervention effects in the same way in every included studies.

The quality of methodology plays an essential role in the strength of evidence: pre-registered protocols, rigorous study design (including application of suitable placebo controls) and adherence to adequate reporting will improve the credibility of findings. None of the included studies performed protocol registration before trial conduct. A weakness of the data set used in the present meta-analysis is that only one study (28) introduced a placebo. The blinding of participants and personnel could be easily compromised due to the low application rate of placebo controls. The remaining RCTs were almost universally not blinded, increasing the risk of bias significantly. Although a perfect imitation of a CHM formula to use as placebo is difficult to devise due to its special color, taste and smell, two main types of placebo composition in the WHO-registered trials of CHM have been commonly employed: 63% of placebo control formulas used excipients (coloring and favoring agents, excluding CHM ingredients) and 37% of them included CHM ingredients (76). Reporting of the characteristics of placebo controls is encouraged. Details such as composition, physical similarity, pharmacologically inert tests, quality control and evaluation methods are recommended. Similarly, information about CHM regimen characteristics should also be detailed, including composition (herbal ingredients), the place of origin, processing method, dosage of the herbs etc. However, four of the included studies did not even list the herbal ingredients. Additionally, information on allocation concealment and blinding of outcome assessment were absent. As the majority of the included studies were published before 2017, i.e., the publication date of CONSORT Extension for Chinese Herbal Medicine Formulas 2017 (77), future study design and reporting methods could be improved by following the CONSORT statement.

## Conclusion

Noting the limitation of quality and quantity of the results, this meta-analysis demonstrates that a combination of CHM and other therapies administered in CVD studies may attenuate inflammatory biomarker levels, especially hs-CRP, IL-6, TNF-α, MCP-1, sICAM-1 and sVCAM-1. Dan shen, Huang qi and Gan cao were the most frequently used herbs in the included formulas with anti-inflammatory effects, which are worth exploring further in the context of CVD in settings of chronic inflammatory conditions including HIV infection. Rigorously-conducted trials and adequate reporting following published standards are needed to provide more robust findings.

## Data availability statement

The original contributions presented in this study are included in the article/Supplementary material, further inquiries can be directed to the corresponding author.

## Author contributions

AJ, AZ, and ML conceptualized the review. ML performed the search. ML and IZ performed manuscript screening, data mining, and data analysis. AZ discussed disagreement. ML took the lead in writing the manuscript and wrote the original draft. JT, AH, AZ, and AJ provided editing and feedback on each version of the manuscript. All authors approved the final version of the manuscript accepted for publication.

## Abbreviations

AIDS: acquired immunodeficiency syndrome
CHM: Chinese herbal medicine
CI: confidence interval
CVD: cardiovascular disease
HIV: human immunodeficiency virus
hs-CRP: high sensitivity c-reactive protein
sICAM-1: soluble intercellular adhesion molecule-1
IL: interleukin
MCP-1: monocyte chemotactic protein-1
MD: mean differences
PWH: people with HIV
RCT: randomized controlled trial
SMD: standardized mean difference
TNF: tumor necrosis factor
sVCAM-1: soluble vascular cell adhesion molecule 1
WM: western medicine.
