# Supplementary material for: Effects and safety of Chinese herbal medicine on inflammatory biomarkers in cardiovascular diseases: A systematic review and meta-analysis of randomized controlled trials
Source: Front Cardiovasc Med. 2022 Aug 16;9:922497. doi: 10.3389/fcvm.2022.922497 (PMC9425052; doi:10.3389/fcvm.2022.922497)
Supplement: Supplementary file 1 [file Data_Sheet_1.docx]

Supplementary Material

# Supplementary Tables

**Table S1****.** Search strategies of clinical studies and experimental studies in PubMed database

| Terms | Search | Query |
| --- | --- | --- |
| Clinical studies | | |
| Interventions | #1 | Search herb |
|  | #2 | Search herbal medicine |
|  | #3 | Search Chinese medicine |
|  | #4 | Search traditional Chinese medicine |
|  | #5 | Search herbal formula* |
|  | #6 | #1 OR #2 OR #3 OR #4 OR #5 |
| Conditions | #7 | Search vascular disease* |
|  | #8 | Search arterial occlusive disease* |
|  | #9 | Search hypertension |
|  | #10 | Search peripheral vascular disease* |
|  | #11 | Search myocardial ischemia |
|  | #12 | Search cardiac arrest |
|  | #13 | Search heart arrest |
|  | #14 | Search cardiovascular disease* |
|  | #15 | Search ischaemic heart disease* |
|  | #16 | Search coronary artery disease* |
|  | #17 | Search atherosclerosis |
|  | #18 | Search inflammat* |
|  | #19 | Search anti-inflammat* |
|  | #20 | #7 OR #8 OR #9 OR #10 OR #11 OR #12 OR #13 OR #14 OR #15 OR #16 OR #17 OR #18 OR #19 |
| #21 | | #6 AND #20 |
| Filter applied | | Randomized controlled trial |

**Table S2.** Interventions of included comparisons from identified randomized controlled trials of Chinese herbal medicine on cardiovascular condition

| Study ID | Control Group | |  | Treatment Group |  |  |  |
| --- | --- | --- | --- | --- | --- | --- | --- |
|  | Intervention | Details | Intervention | Formula Name | Ingredients | Administration | Form |
| **CHM plus WM versus CHM placebo plus WM** | | | | | | | |
| Li et al., 2009 | WM + placebo | **Placebo:** NS  **WM:** nimodipine capsules, 1 capsule, tid | CHM + WM | Jiang Ya Capsule | Chuan niu xi, Di long, Hai zao, Tian ma, Chuan xiong etc. | 4 capsules (each capsule contains 0.3 g, containing a total of 0.54 g of crude drug) | Capsule |
| **CHM plus co-intervention versus the same co-intervention** | | | | | | | |
| Chen, 2015 | Routine | Oxygen intervention;  routine administration of vasodilators, diuretics, β-blockers, and symptomatic support drugs such as trimetazidine and avastatin (20 mg, tid) | CHM + Routine | Yi Xin Tang | Dang shen, Huang qi, Wu wei zi, Dan shen, Ma huang, Chuan xiong, Chi shao, Gan cao | The prescription was soaked in 1500 ml water for 30 min, then decocted in water to 300 ml, and divided into 2 bags. One bag each time, bid | Decoction |
| Chen & Cai, 2019 | Routine | Patients with grade 2 hypertension should take medication to control blood pressure. Antihypertensive drugs should be administered in small doses, with priority given to long-acting agents, combined use, and individualized treatment. CCB, ACEI, β-blocker, diuretics and other antihypertensive drugs were used to control the blood pressure of the treatment group and control group under 140/90mmhg according to the blood pressure change | CHM + Routine | Modified Xiao Xian Xiong Tang | Gua lou, Dan nan xing, Ban xia, Huang lian, Zhu ru etc. | 200ml/dose, bid | Decoction |
| Chen et al., 2008 | Routine | Active control of the primary disease, hypertension patients were hypotensive treatment (diuretics, angiotensin converting enzyme inhibitors, β-blockers), fluvastatin 40mg, qd | CHM + Routine | Yi Qi Huo Xue Hua Yu Tong Luo Fang | Huang qi, Dang gui, Dan shen, Sang shen, Di long, Ze xie, Shi chang pu, Cha shu gen | 140ml/bag, 1 bag, taken before breakfast and dinner. | Decoction |
| Dai, 2020 | WM + Lifestyle | **WM:** hydrochloride tablets, 10 mg, qd; amlodipine tablets, 5 mg qd.  **Lifestyle:** guidance on optimizing diet structure, increasing exercise, improving mood, and reducing weight. | CHM + WM + Lifestyle | Yang Gan Yi Shui Fang | Gou qi zi, Tu si zi, Dan shen, Huang qi, Huai niu xi | 1 dose/day, decocted in 300ml water, divided into 2 sub-doses taken once in the morning and once in the evening | Decoction |
| Ding & Hu, 2020 | WM + Lifestyle | **WM**: aspirin enteric-coated tablets, 100 mg, qd; atorvastatin calcium tablets, 20 mg, qd; metoprolol tartrate tablets, 12.5 mg, bid; isosorbide mononitrate sustained-release tablets, 20 mg, bid  **Lifestyle**: limit smoking and alcohol, low salt and low fat | CHM + WM + Lifestyle | Shu Gan Qing Zhi Fang | Chai hu, Dang gui, Shan zha, Suan zao ren, Jiao gu lan, Gan cao | All the above medicines with warm water, 100 ml/ time, 3 times /day | NA |
| Fan et al., 2012 | WM | Amlodipine besylate tablets 5mg, 1 tablet, qd | CHM + WM | Sou Feng Tong Luo Fang | Gui ban, Jiang cao, Di long, Huang qi, Dan shen, Dang gui, Sang shen, Cha shu gen, Shi chang pu, Ze xie etc. | 100ml/ bag, 1 bag, bid | Decoction |
| Huang et al., 2011 | WM | Atorvastatin 20 mg/ day | CHM + WM | Huo Xue Li Shuo Zhong Yao Fu Fang | Huang qi, Dan shen, Chong wei zi, Che qian zi, Xia ku cao | bid | Herbal extraction |
| Huang, Qian & Li, 2010 | WM | Simvastatin 20mg /day | CHM + WM | Zhi Long Huo Xue Fang | Shui zhi, Di long | bid | Herbal extraction |
| Jia, 2016 | WM + Routine | **Routine treatment:** antiplatelet therapy, hypotensive and hypoglycemic routine therapy  **WM:** atorvastatin, 10 mg, bid, after meals | CHM + WM + Routine | Na | Huang qi, Dan shen, Yu jin, Bai zhu, Ren shen, Hong hua, Ge gen Gui zhi, Dang gui | bid | Decoction |
| Jin, Zhu & Guo, 2016 | WM + Routine + Lifestyle | **Lifestyle:** all patients were advised to follow a strict diet;  **Routine:** take their concomitant oral medication;  **WM:** simvastatin capsules. 20mg, qd, at night | CHM + WM + Routine + Lifestyle | Shen Qi Rou Mai He Ji | Pu gong ying, Da huang, Sheng di huang, Huang qi, Ren shen, Gui zhi, Jin yin hua, Bai shao, Chuan xiong, Sheng shan zha, Ban xia, Zhi shou wu, Gan cao | 100 ml/dose, with one dose in the morning and one dose in the evening | Decoction |
| Li & Long, 2013 | WM + Routine | **Routine:** antiplatelets, beta blockers and nitrates  **WM:** atorvastatin 20 mg, qd, half an hour after dinner | CHM + WM + Routine | Yi Qi Wen Yang Huo Xue Fang | Ren shen, Huang qi, Dan shen, Hong hua, Yu jin, Bai zhu, Gui zhi, Ge gen, Dang gui | 150 ml, bid | Decoction |
| Li et al., 2017 | Routine | ARB + CCB scheme (potassium losartan, valsartan, irbesartan, etc., for ARB, benazepril and perdolopril for ACEI, and amlodipine and felodipine etc., for CCB) | CHM + Routine | Zi Shen Qing Gan Fang | Gui zhen cao, Zhi he shou wu, Shan yu rou, Xuan shen, Ze xie, Chuan niu xi | 10g/bag, 10g, tid | Granule |
| Li, Zhao & Wang, 2021 | WM | Atorvastatin calcium tablets (10 mg/tablet), the initial dose is 10 mg/time, qd, followed by 20 mg/time, qd; Aspirin enteric sol sac (0.3 g/capsule) ,0.1 g/time, qd | CHM+WM | Hua Ban Fang | Chuan xiong, Dan shen, Ze xie, Fu ling, Huang qi, Ge gen, Dang shen, Bai zhu, Tian ma, Dang gui, Chen pi, Shi chang pu, Gan cao, Yin xing ye, Huang lian | 1 dose/day (300 mL), with one dose in the morning and one dose in the evening | Decoction |
| Liu, 2018 | WM + Routine + Lifestyle | **Con:** Antiplatelet, antihypertensive and hypoglycemic routine therapy  **WM:** atorvastatin, taken orally after meals. The initial dose was 10 mg/ time and 1 time/d. The maintenance dose was 30 mg/ time and 1 time /d;  **Lifestyle:** standard low cholesterol diet | CHM + WM + Routine + Lifestyle | Na | Dan shen, Bai zhu, Yu jin, Hong hua, Gui zhi, Ren shen, Dang gui, Huang qi | Each dose was about 150ml, taken orally, 1 dose/time, 2 times/day | Decoction |
| Liu, Chen & Li, 2019 | Routine | Aspirin, 0.1g, qd; Lipitor, 20mg, qd, at night | CHM + Routine | Mixture Of Single Herbs | Dan shen, Chuan xiong | 1 packet in the morning and 1 packet in the evening | Granule |
| Ma et al., 2019 | WM + Lifestyle | **WM:** Irbesartan tablets (0. 15 g/tablet), 1 tablet, qd, in the morning; **Lifestyle:** health education on hypertension and improved their lifestyle behaviors before receiving medication, including weight control, calcium and potassium supplementation, sodium intake reduction, fat intake reduction, alcohol restriction and increased exercise. | CHM + WM + Lifestyle | Ban Xia Bai Zhu Tian Ma Tang | Bai zhu, Ju hong, Ban xia, Tian ma, Fu ling, Da zao, Sheng jiang, Gan cao | Used 400 ml boiled water to brew one dose of granules. 200 ml/time, bid, in the morning and night | Granule |
| Meng et al., 2016 | Routine | **Routine:** Routine antihypertensive therapy  **WM:** simvastatin tablets, 20mg qd | CHM + Routine | Fu Fang Dan Shen Di Wan | NA | 270 mg, tid | Pill |
|  | WM + Routine |  | CHM + WM + Routine |  |  |  |  |
| Qian et al., 2013 | WM | Amlodipine (5 mg/tablet), 5 mg/time /d; valsartan (80 mg/tablet), 80 mg/dose /d | CHM + WM | Jiang Zhi Kang Yang Hua He Ji | He shou wu, Shan zha, Lian qiao, Ge gen | 20 ml, tid | Decoction |
| Tian, 2018 | WM + Lifestyle | **Lifestyle:** implemented quit smoking and alcohol, control salt intake, etc.;  **WM:** oral amlodipine besylate tablets (5mg/tablets), 1 tablet, qd, if the antihypertensive effect is not satisfactory, add benazepril hydrochloride tablets (5mg/tablet), 1 tablet, qd. Adjusted the dose according to patient tolerance and blood pressure. | CHM + WM + Lifestyle | Modified Qing Xuan Jiang Ya Tang | Huang qin, Ku ding Tea, Sheng di huang, Chuan niu xi, Ju hua, Shi hu, Du zhong, Ye jiao teng, Tian ma, Gou teng, Sang ye, Gui ban | 200ml, bid | Decoction |
| Wan & Li, 2020 | WM | Isosorbide mononitrate, 40 mg, qd; atorvastatin calcium, 30 mg, qd | CHM + WM | Huo Xue Tong Lup Fang | Tao ren, Dan shen, Chuan xiong, Chi shao, Hong hua, Dang gui, Shui zhi, Di long | Decocted with 400 ml water, 1 dose /d, taken warm in the morning and evening | Decoction |
| Wang et al., 2016 | Routine | Levamlodipine benzenesulfonic acid tablets, 2.5 mg, qd, if after 2-week treatment, diastolic blood pressure was still higher than the upper limit, increase the dose to 5 mg, qd. If the blood pressure control was still not ideal, add drugs of other hypotensive mechanisms | CHM + Routine | Ping Gan Qian Yang Fang | Zhu xian cao, Xia ku cao, Shi jue ming, Huai hua, Tian ma, Huang qin, Dan shen, Chuan niu xi, Sang ji sheng, Che qian zi | 150ml, bid | Decoction |
| Xie et al., 2019 | WM | Simvastatin, 20 mg, qd | CHM + WM | Shen Qi Mai Xin Tong Capsule | NA | 2 capsules, tid | Capsule |
| Xie, Wu & Kong, 2018 | Routine | Amlodipine besylate tablets, 5 mg qd. The target of pressure control is systolic pressure < 140mmHg. Drug usage and dosage adjustment plan: the drug usage and dosage should be adjusted once a week. If the patient's blood pressure still failed to reach the target of pressure control after taking the drug, benapril should be given 10mg, qd. If after 1-week treatment, blood pressure was still not up to patient’s control target, continue to offer hydrochlorothiazide in 12.5 mg, qd. The maximum dosage of amlodipine was 10mg, qd. The maximum dosage of Benazepril was 20mg, qd. The maximum dosage of hydrochlorothiazide was 25mg, qd. If after adding to the maximum dose, patient’s blood pressure still failed to reach the goal, stop increasing medication dose. | CHM + Routine | Self-Designed Yi Qi Hua Tan Fang | Huang qi, Dai zhe shi, Shi chang pu, Dang shen, Fu ling, Chen pi, Fa ban xia, Shi jue ming, Bai zhu, Gan cao | Decocted in 800ml water to 150ml, qd | Decoction |
| Xiong & Zhu, 2021 | WM | Amlodipine besylate tablets, 5 mg, qd, in the morning. | CHM+WM | Tiao Zhi Jiang Ya Fang | Fa ban xia, Huang lian, Chuan xiong, Tian ma, Shui zhi, Gou teng, Lai fu zi, Gui zhen cao, Dan shen | 30 pills / time, tid. | Pill |
| Xu et al,. 2021 | WM | Amlodipine, 5mg/d; atorvastatin calcium tablets 10mg/d | CHM+WM | Qian Yang Yu Yin Granule | Gui zhen cao, He shou wu, Shan yu rou, Xuan shen, Niu xi, Ze xie | 10g per sachet, tid | Granule |
| Yang & Huang, 2021 | WM | Amlodipine atorvastatin calcium tablets, a starting dose of 5 mg and a maximum daily dose of 10 mg, according to the patient's condition | CHM + WM | Xue Fu Zhu Yu Capsule | NA | 3 pills, tid | Capsule |
| Yang & Li, 2016 | Routine + Lifestyle | **Routine:** Lipid regulation, blood pressure reduction, anti-platelet aggregation, etc  **Lifestyle:** Lifestyle improvement (low-salt and low-fat diet, morning exercise, good daily habits, etc.) | CHM + Routine + Lifestyle | Bu Shen Jie Yu Tang | Sang ji sheng, Nv zhen zi, Yin yang huo, Chai hu, Dang gui, Chi shao, Fu ling, Bai zhu, Bo he, Tian ma, Gou teng, Niu xi, Gan cao | 200ml, bid, taken in the morning and evening | Decoction |
| Yao, Zhang & Zeng, 2021 | WM + Lifestyle | Atorvastatin, 20 mg, qd, taken at bedtime; amlodipine benzenesulfonate tablets, 5 mg, qd; aspirin, 0.1 g, qd | CHM+WM + Lifestyle | Yi Qi Huo Xue Tong Mai Tang | Huang qi, Chuan xiong, Dang shen, Ge gen, Hong hua, Dan shen, San qi, Gan cao | 500 ml/day, taken after breakfast and dinner | Decoction |
| Yu, 2012 | Routine | Aspirin, 100mg/day; perindopril 4-8mg/day; metoprolol tartrate 12.5 ~ 25mg, qd; atorvastatin, 20mg, bid; valsartan, 100mg, qd | CHM + Routine | Single Herb | Da huang | 3g/ bag per day | Granule |
| Zeng, Tang & Qi, 2017 | Lifestyle | (1) Reasonable diet; (2) control mass; (3) give up smoking and drinking; (4) proper physical exercise; (5) reduce psychological pressure, keep the mood happy | CHM + Lifestyle | Gan Lu Xiao Du Dan | Hua shi, Huang qin, Mian yin chen, Shi chang pu, Zhe bei mu, Chuan mu tong, Huo xiang, Lian qiao, Bai dou kou, Bo he, She gan, Gan cao | 150ml, bid, take medication for 2 weeks every month | Decoction |
| Zhang et al., 2012 | WM | Nifedipine sustained release tablets, 10mg, q12h; If not, the dose can be increased | CHM + WM | Ci Zhu Te Jiang Capsule | NA | 4 capsules, tid | Capsule |
| Zhang et al., 2014 | Routine | Fosinopril sodium, 10 mg, qd (or valsartan, 80 mg, qd). Calcium antagonists (CCB's) or diuretics were added if the antihypertensive effect was poor. Aspirin enteric-coated tablets, 0.1 g, qd | CHM + Routine | Shu Nao Xin Di Wan | NA | 4 pills, tid | Pill |
| Zheng et al., 2009 | Routine | Statins (simvastatin, atorvastatin, etc.) | CHM + Routine (T_1_: Huo Xue) | Dan Qi Pian | Dan shen, San qi | 3 tablets (0.3g/ tablet), tid | Tablet |
|  |  |  | CHM + Routine (T_2_: Huo Xue Jie Du) | Xin Qing Ning Pian | Da huang (Shu) | 3 tablets (0.3g/ tablet), tid | Tablet |
| Zhou, 2021 | WM | Nifedipine extended-release tablets, 10mg, bid; atorvastatin calcium, 20 mg, qd, taken before bedtime | CHM+WM | Tong Mai Jie Du Fang | San qi, Pu huang, Dan shen, Gua lou pi, Che qian zi, Du Zhong, Gou qi zi, Huang jing, Fu ling, Ci ji li | 1 dose, qd, taken 30 minutes before breakfast and dinner | Decoction |
| Zhu et al., 2014 | Routine | Standard treatment for CHD, including nitrate drugs such as isosorbide mononitrate tablets, 20 mg, bid; isosorbide dinitrate, 5-10 mg, tid; and nitroglycerin according to the individual’s condition; aspirin 100-300 mg, qd; statins for regulating lipids such as atorvastatin calcium tablets 10-20 mg, qd; and fluvastatin sodium capsules, 40 mg, qd, at night; angiotensin-converting enzyme inhibitor (Benazepril tablets), 10 mg, qd; or peduopril tablets, 4 mg, qd. For patients with no contraindications, β-receptor blockers (Metoprolol tablets) 6.25-100 mg, bid; bisoprolol tablets 2.5-5.0 mg, qd were used for long-term treatment over the whole course of the study. | CHM + Routine | Liandouqingmai Recipe | Lianqiao (Fructus Forsythiae) 15 g, Huanglian (Rhizoma Coptidis) 3 g, Yeliaodou (Glycine soja Sieb) 15 g, Chishao (Radix Paeoniae) 10 g, Laifuzi (Semen Raphani) 10 g, and so on | 250 ml, bid | Decoction |
| Zhu et al., 2019 | Routine | Atorvastatin calcium tablets, 1 tablet, qd, at night; aspirin enteric-coated tablets, 1 tablet, qd, at night; vasodilators, β-blockers and other symptomatic treatment. | CHM + Routine | Lu Huang Granule | Hu zhang, Lou lu, Zhi he shou wu, Guang jing, Jiang huang, Hong hua | 1 dose per day, decocted in water, bid, in the morning and evening | Granule |
| Zuo, Zhang & Gu, 2014 | Routine | Felodipine sustained release tablets, 5mg, qd. If the blood pressure did not drop below 140/90mmhg after 2-week treatment, benazepril was taken 10mg, qd | CHM + Routine | Sang Ji He Ji | Sang ye, Bai ji li, Ju hua, Zhi ban xia, Chao jue ming zi, Zhu ru (fried with Jiang zhi), Chen pi, Fu ling, Gu jing cao, Zhi ke, Gan cao | 20ml/ time tid | Decoction |

Abbreviations: ACEI, angiotensin converting enzyme inhibitors; ARB, angiotensin II receptor blockers; bid, twice a day; CCB, calcium channel blockers; CHM, Chinese herbal medicine; NA, not applicable, the formula ingredients were not provided by the paper or identified from Zhong Yi Fang Ji Da Ci Dian; NS, not specified; Routine, routine treatment; qd, once a day; tid, three times a day; WM, western medicine. Routine treatment: where the only the categories (e.g. beta-blockers) of the drugs were provided but did not specified drug name and/or dosage; routine care such as oxygen intervention were included in the intervention; or authors claimed to be a routine/standard treatment.

**Table S3.** Studies reported parameters with unmatched baseline values

| Study ID | Parameters with unmatched baseline values | Comparisons | Baseline meta-analysis |
| --- | --- | --- | --- |
| Liu, 2018 | IL-6 | CHM + WM + Routine + Lifestyle vs WM + Routine + Lifestyle | MD -0.39, 95% CI -0.57 to -0.21 |
| Xie, Wu & Kong, 2018 | IL-6 | CHM + Routine vs Routine | MD 0.02, 95% CI 0.00 to 0.04 |
| Zheng et al., 2009 | hs-CRP | CHM + Routine (T_2_: Huo Xue Jie Du) vs Routine | MD 5.94, 95% CI 2.79 to 9.09 |
| Zhu et al., 2014 | hs-CRP | CHM + Routine vs Routine | MD -1.84, 95% CI -3.51 to -0.17 |

Abbreviations: CHM, Chinese herbal medicine; CI, confidence intervals; hs-CRP, hyper sensitivity C-reactive protein; IL, interleukin; MD, mean difference; T, treatment group; WM, western medicine.

**Table S4.** Adverse events

| Study ID | Treatment groups | Control groups |
| --- | --- | --- |
| Chen, 2015 | Not assessed | Not assessed |
| Chen & Cai, 2019 | All the included cases in this study underwent routine examinations of liver and kidney function, blood, urine and stool before and after treatment, and no abnormalities were found. No other adverse events were observed during treatment | |
| Chen et al., 2008 | No obvious adverse events were found in the treatment group before and after treatment | ALT increased in 1 case in the control group after treatment, which returned to normal after 2 weeks of withdrawal |
| Dai, 2020 | Not assessed | Not assessed |
| Ding & Hu, 2020 | Not assessed | Not assessed |
| Fan et al., 2012 | No obvious adverse events were found in the treatment group before and after treatment | ALT increased in 1 patient in the control group after treatment and recovered normally after 2 weeks of withdrawal. |
| Huang et al., 2011 | Not assessed | Not assessed |
| Huang, Qian & Li, 2010 | Not assessed | Not assessed |
| Jia, 2016 | Not assessed | Not assessed |
| Jin, Zhu & Guo, 2016 | There was no abnormal renal function, blood, urine or stool routine in treatment group during treatment. | There was no abnormal renal function, blood, urine or stool routine in treatment group during treatment.  One patient in the control group showed elevated glutamic-oxalacetic transaminase, and liver function returned to normal 2 weeks after drug withdrawal. |
| Li & Long, 2013 | Not assessed | Not assessed |
| Li, Zhao & Wang, 2021 | Gastrointestinal track reaction (n=2), liver damage (n=1), dizziness (n=1), dysgeusia (n=1). (P<0.01) | Gastrointestinal track reaction (n=5), liver damage (n=3), dizziness (n=6), dysgeusia (n=4) |
| Li et al., 2009 | All the included groups had good safety, and there were no significant changes in blood routine, urine routine and ECG before and after treatment. The indexes of liver and kidney function (ALT, Cr, BUN) before and after treatment in the included groups were all within the normal range, and there was no case whose fluctuation was more than 50% of the normal value before and after treatment.  During the observation, one patient showed facial flushing and dizziness, and the symptoms disappeared spontaneously after continuing to take the medicine for one week. (The author did not mention which group the patient is from) | |
| Li et al., 2017 | Alanine aminotransferase (MD -0.23, 95% CI -7.43 to 6.97); ceramic oxalacetic transaminase (MD 0.17, 95% CI -3.30 to 3.64); blood urea nitrogen (MD -0.50, 95% CI -1.44 to 0.44); serum creatinine (MD -2.55, 95% CI -11.88 to 6.78) | |
| Liu, 2018 | Not assessed | Not assessed |
| Liu, Chen & Li, 2019 | Not assessed | Not assessed |
| Ma et al., 2019 | There were no obvious adverse events in both groups during treatment, and all routine examinations were normal. | |
| Meng et al., 2016 | Not assessed | Not assessed |
| Qian et al., 2013 | Not assessed | Not assessed |
| Tian, 2018 | Not assessed | Not assessed |
| Wan & Li, 2020 | Not assessed | Not assessed |
| Wang et al., 2016 | Not assessed | Not assessed |
| Xie et al., 2019 | Not assessed | Not assessed |
| Xie, Wu & Kong, 2018 | Not assessed | Not assessed |
| Xiong & Zhu, 2021 | Not assessed | Not assessed |
| Xu et al., 2021 | There were no obvious adverse events occurred in the two groups during treatment, and there were no obvious changes in vital signs, blood routine, urine routine, electrocardiogram, liver and kidney function before and after treatment | |
| Yang & Huang, 2021 | There were no obvious adverse events in both groups. | |
| Yang & Li, 2016 | There were no serious adverse events in the two groups. | |
| Yao, Zhang & Zeng, 2021 | Gastrointestinal discomfort (n=1), paresthesia (=2), skin rash (n=1). There was no statistically significant difference in the incidence of adverse reactions between the two groups (χ2=340), and the symptoms of patients with adverse reactions were mild, and they were relieved after symptomatic treatment. | Gastrointestinal discomfort (n=2), liver and kidney function impairment (n=1), skin rash (n=3) |
| Yu, 2012 | Not assessed | Not assessed |
| Zeng, Tang & Qi, 2017 | There were no significant changes in blood and urine routine, liver and kidney function in the included groups before and after treatment.  In the treatment group, nausea, vomiting and abdominal distension occurred in 1 case (7.5%). There was no significant difference in the incidence rate of ADR between the two groups (P > 0.05). | There were no significant changes in blood and urine routine, liver and kidney function in the included groups before and after treatment.  Dizziness occurred in 1 case (2.5%.) in control group |
| Zhang et al., 2012 | After treatment, no abnormalities were found in blood routine, urine routine, fecal routine, liver and kidney function and other safety indexes of all patients in both groups.  Headache (n=2), facial flushing (n=3), and ankle edema (n=1), and none of them withdrew from the trial.  The adverse reactions between the two groups were significant (P < 0. 05). | After treatment, no abnormalities were found in blood routine, urine routine, fecal routine, liver and kidney function and other safety indexes of all patients in both groups.  Headache (n=6), facial flushing (n=8), and ankle edema (n=5), among which 1 case quit due to headache intolerance.  The adverse reactions between the two groups were significant (P < 0. 05). |
| Zhang et al., 2014 | Not assessed | Not assessed |
| Zheng et al., 2009 | Liver and kidney functions of all patients in each group were within normal range before and after treatment. One patient (T_2_: Huo Xue Jie Du) reported significantly increased stool frequency after taking the medicine, and the symptoms still existed after the drug was stopped. Then the patient stopped taking the medicine by himself and withdrew from the test. No adverse drug reactions were reported in other patients. | Liver and kidney functions of all patients in each group were within normal range before and after treatment. No adverse drug reactions were reported in other patients. |
| Zhou, 2021 | During the treatment period of the Western medicine group, a patient with transient aspartate aminotransferase was mildly increased by less than 50%. The results of re-examination returned to normal after stopping the drug. The occurrence of adverse events in the groups was not statistically significant (P>0.05). | |
| Zhu et al., 2014 | Not assessed | Not assessed |
| Zhu et al., 2019 | There was no abnormal change in liver and kidney function after treatment in both groups. |  |
| Zuo, Zhang & Gu, 2014 | Not assessed | Not assessed |

# Supplementary Figures


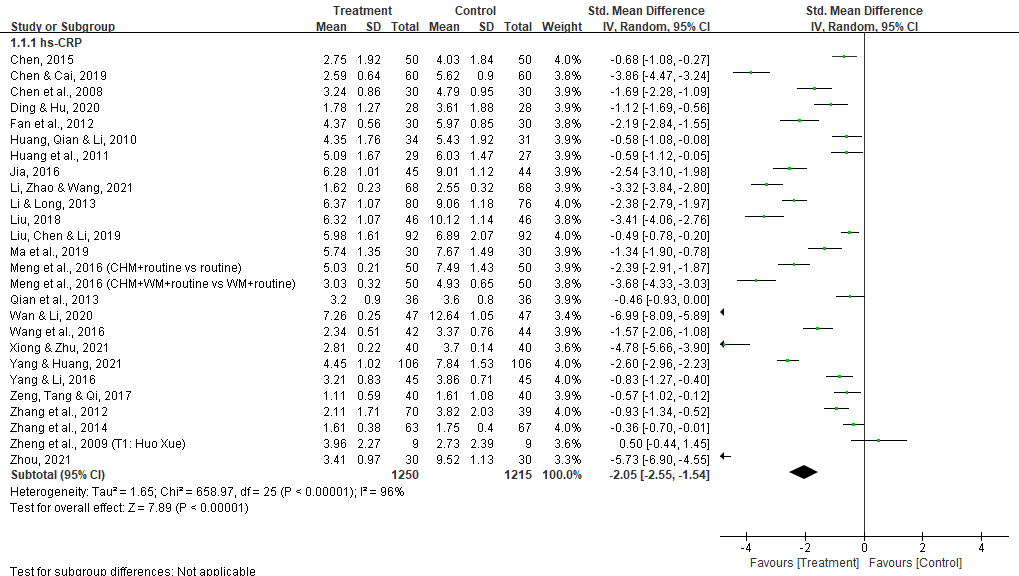


**Figure S1**. Forest plot of included trials investigating the effect of hs-CRP


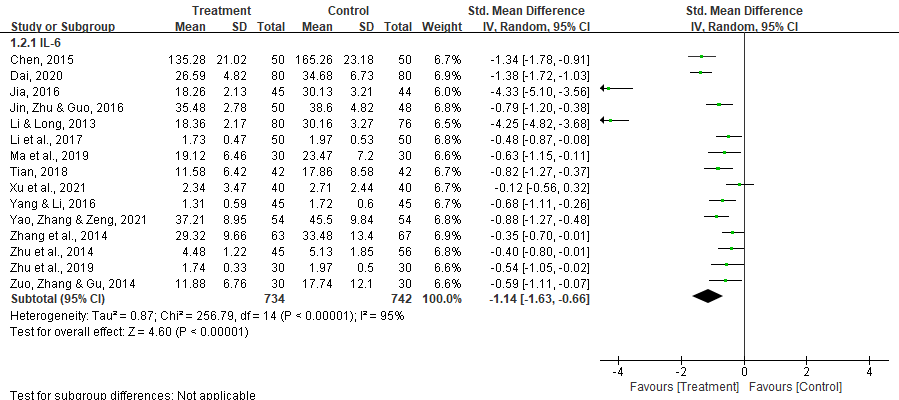


**Figure S2.** Forest plot of included trials investigating the effect of IL-6


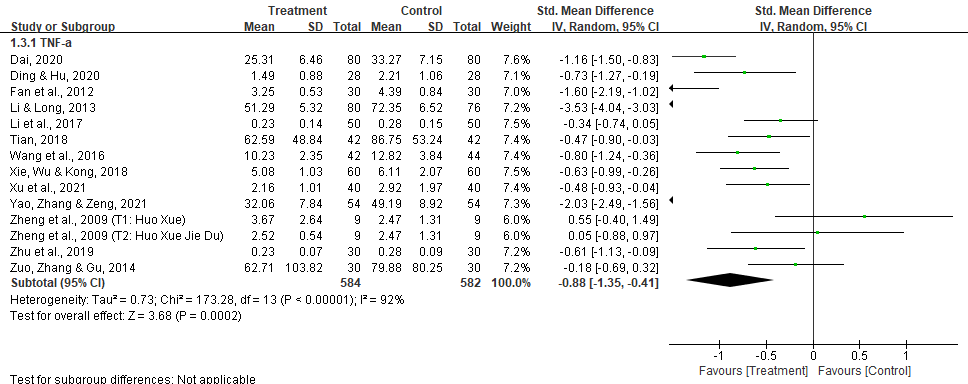


**Figure S3.** Forest plot of included trials investigating the effect of TNF-α


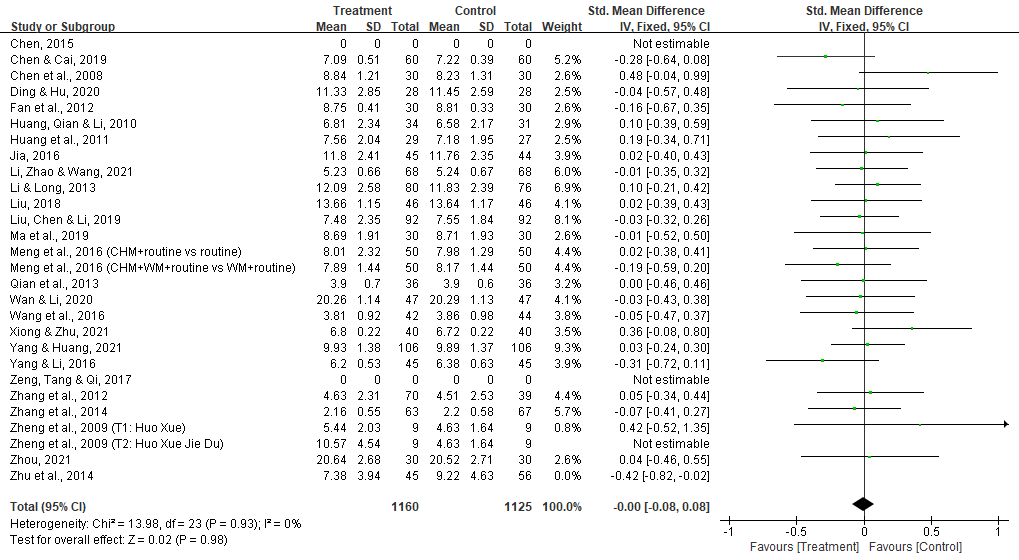


**Figure S4.** Forest plot of hs-CRP between two groups at the baseline. Notes: two comparisons were incomparable at the baseline (Zheng et al., 2009, T2: Huo Xue Jie Du, MD 5.94, 95% CI 2.79 to 9.09 and Zhu et al., 2014, MD -1.84, 95% CI -3.51 to -0.17)


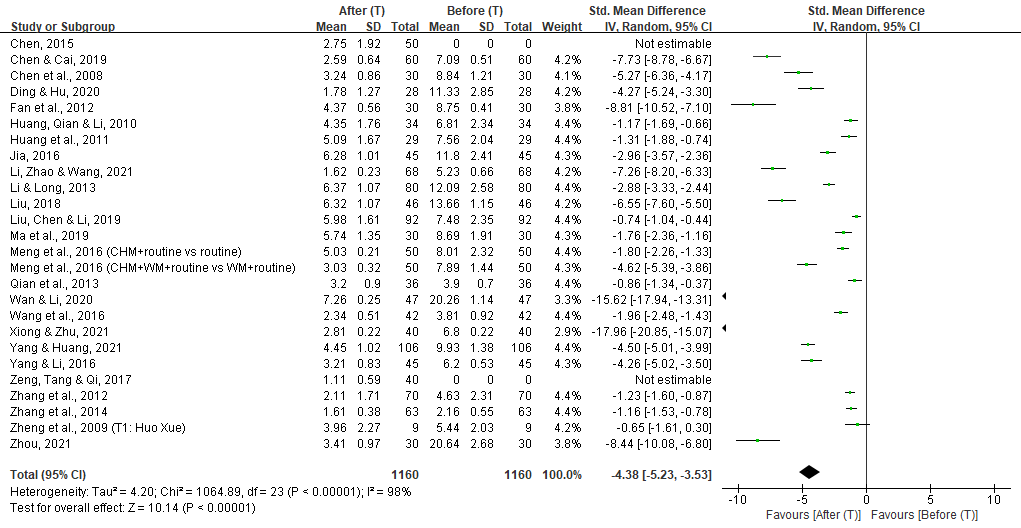


**Figure S5.** Forest plot of hs-CRP in the treatment group (before and after treatment)


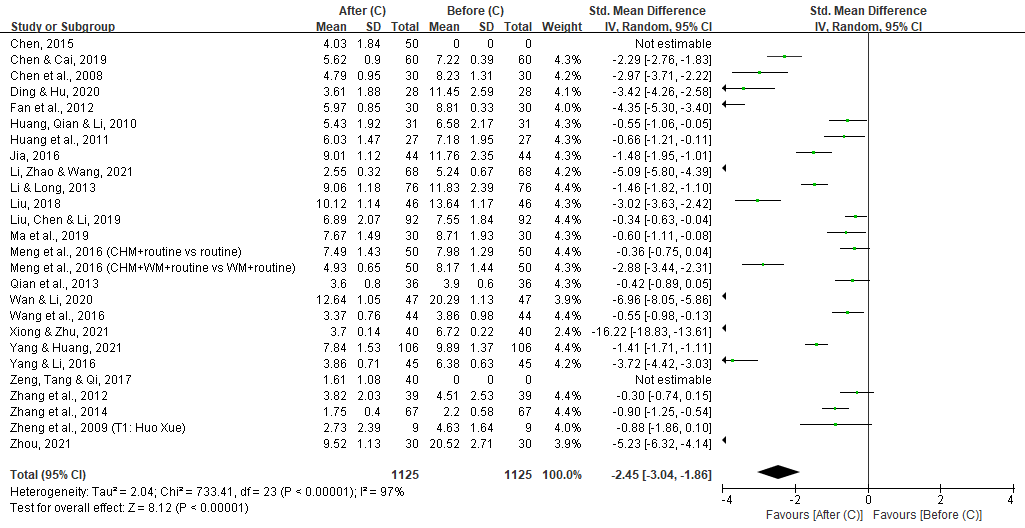


**Figure S6.** Forest plot of hs-CRP in the control group (before and after treatment)


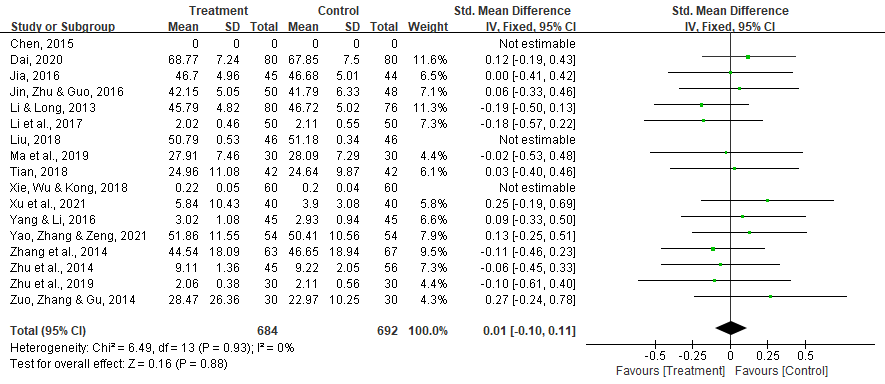


**Figure S7.** Forest plot of IL-6 between two groups at the baseline. Notes: two comparisons were incomparable at the baseline (Liu, 2018, MD -0.39, 95% CI -0.57 to -0.21 and Xie, Wu & Kong, 2018, MD 0.02, 95% CI 0.00 to 0.04).


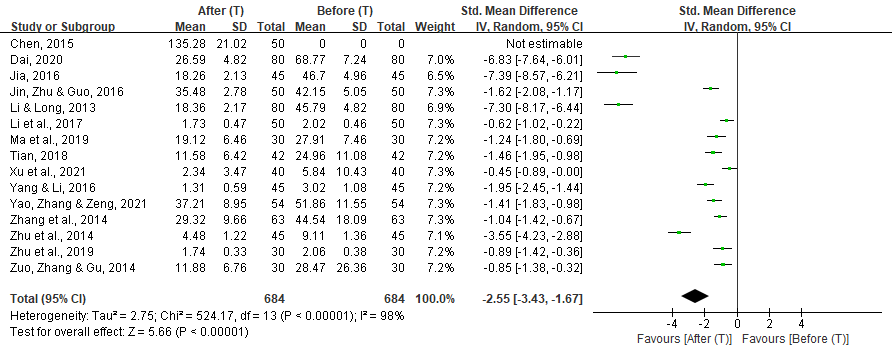


**Figure S8.** Forest plot of IL-6 in the treatment group (before and after treatment)


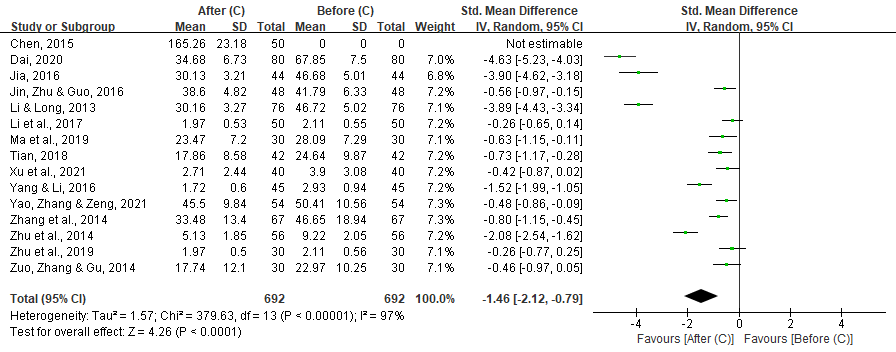


**Figure S9.** Forest plot of IL-6 in the control group (before and after treatment)


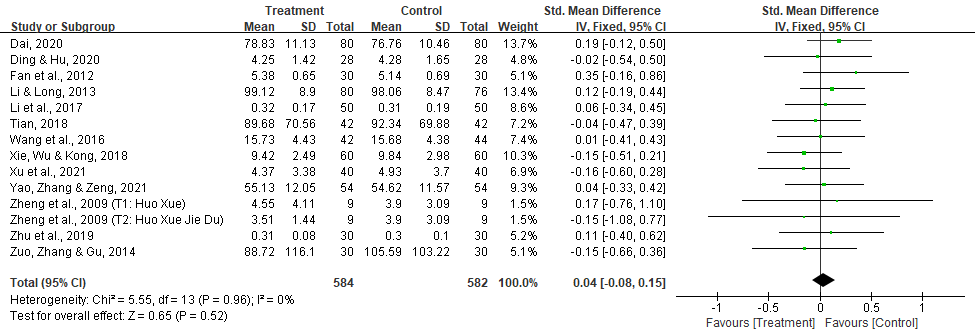


**Figure S10.** Forest plot of TNF-α between two groups at the baseline


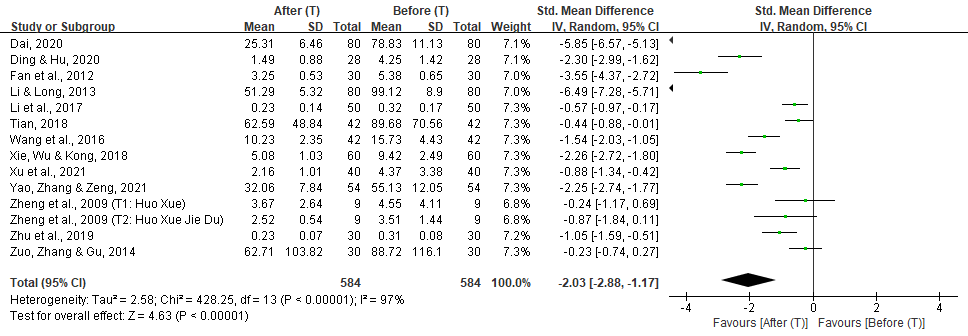


**Figure S11.** Forest plot of TNF-α in the treatment group (before and after treatment)


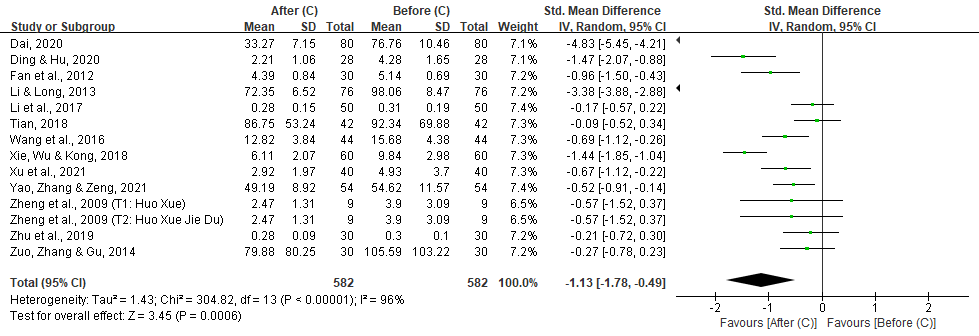


**Figure S12.** Forest plot of TNF-α in the control group (before and after treatment)


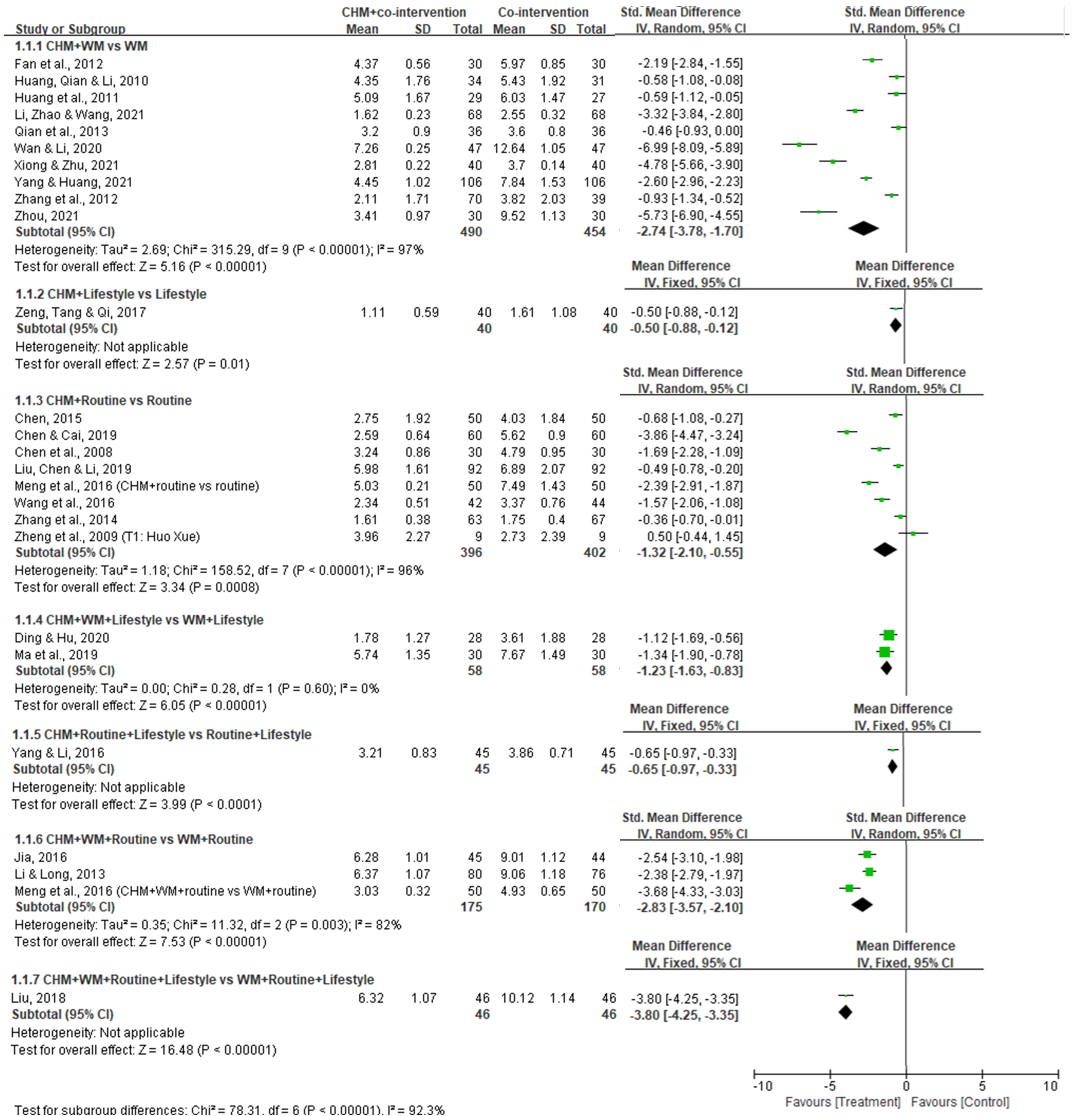


**Figure S13.** Subgroup analysis of hs-CRP when comparing different co-interventions

**
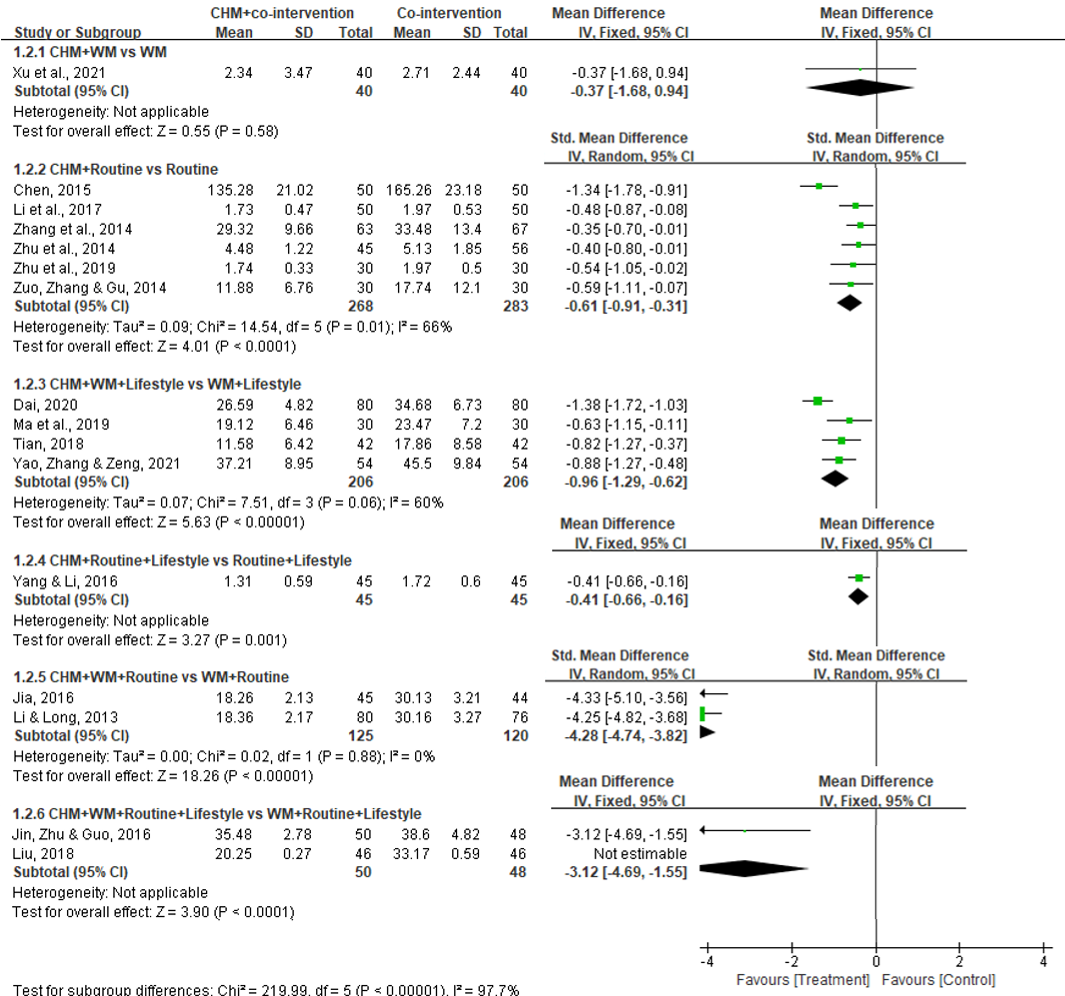
Figure S14.** Subgroup analysis of IL-6 when comparing different co-interventions

**
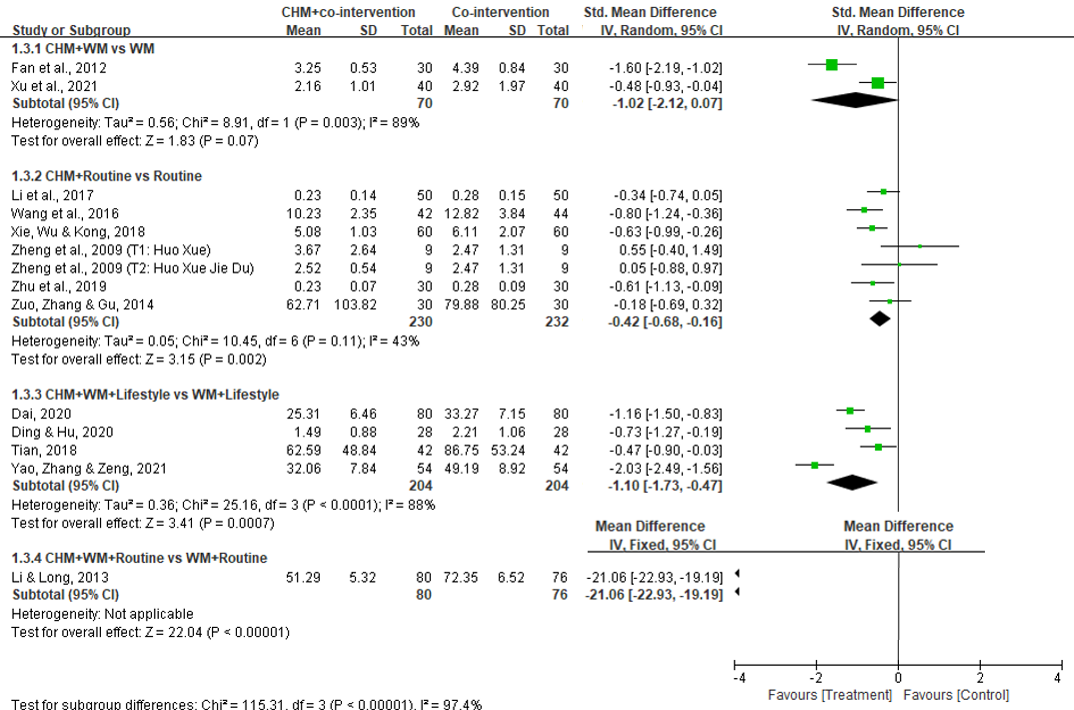
**

**Figure S15.** Subgroup analysis of TNF-α when comparing different co-interventions

**
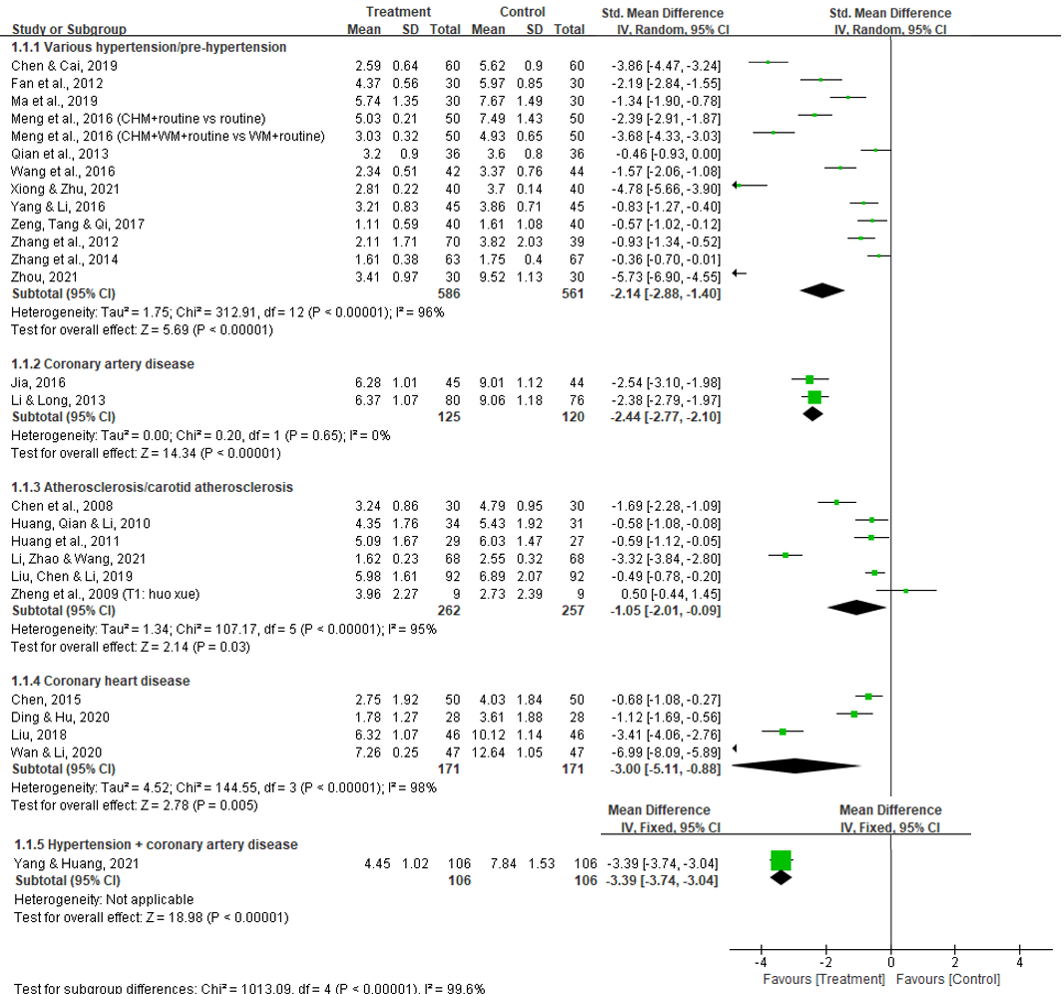
**

**Figure S16.** Subgroup analysis of hs-CRP when comparing different conditions

**
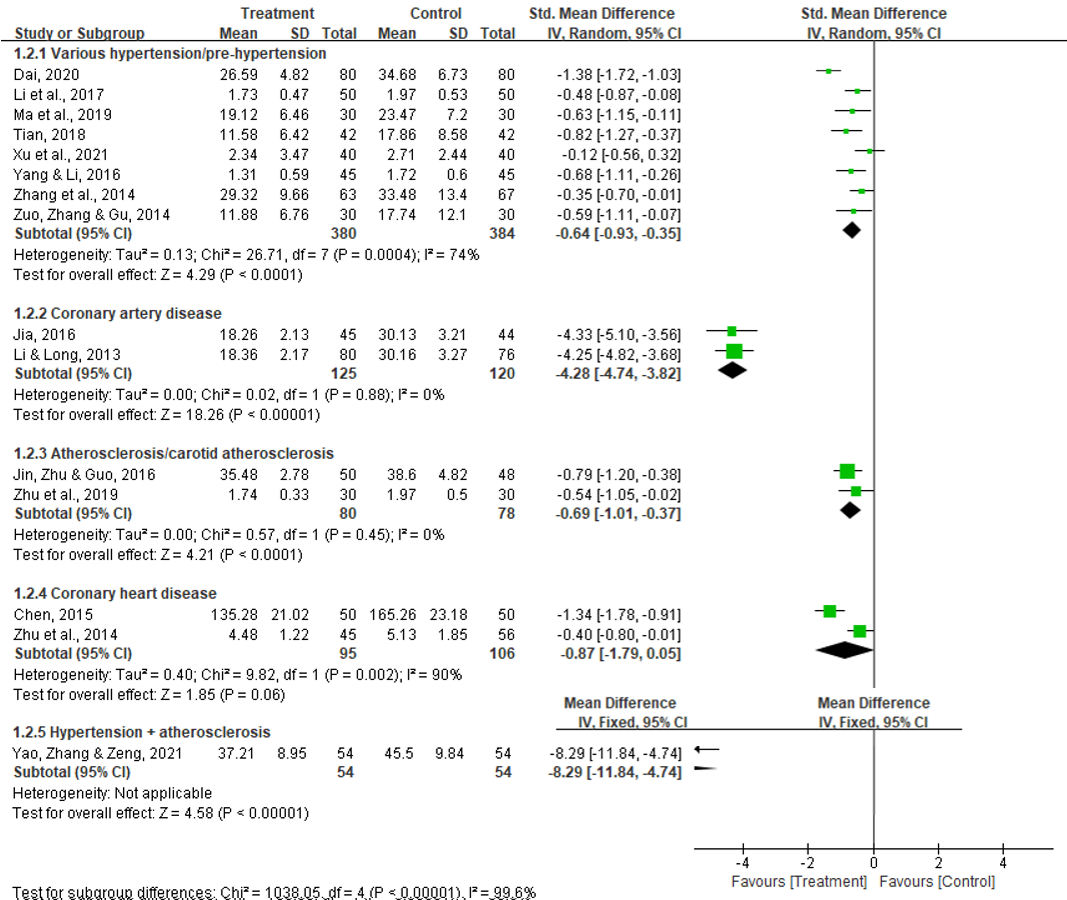
Figure S17.** Subgroup analysis of IL-6 when comparing different conditions

**
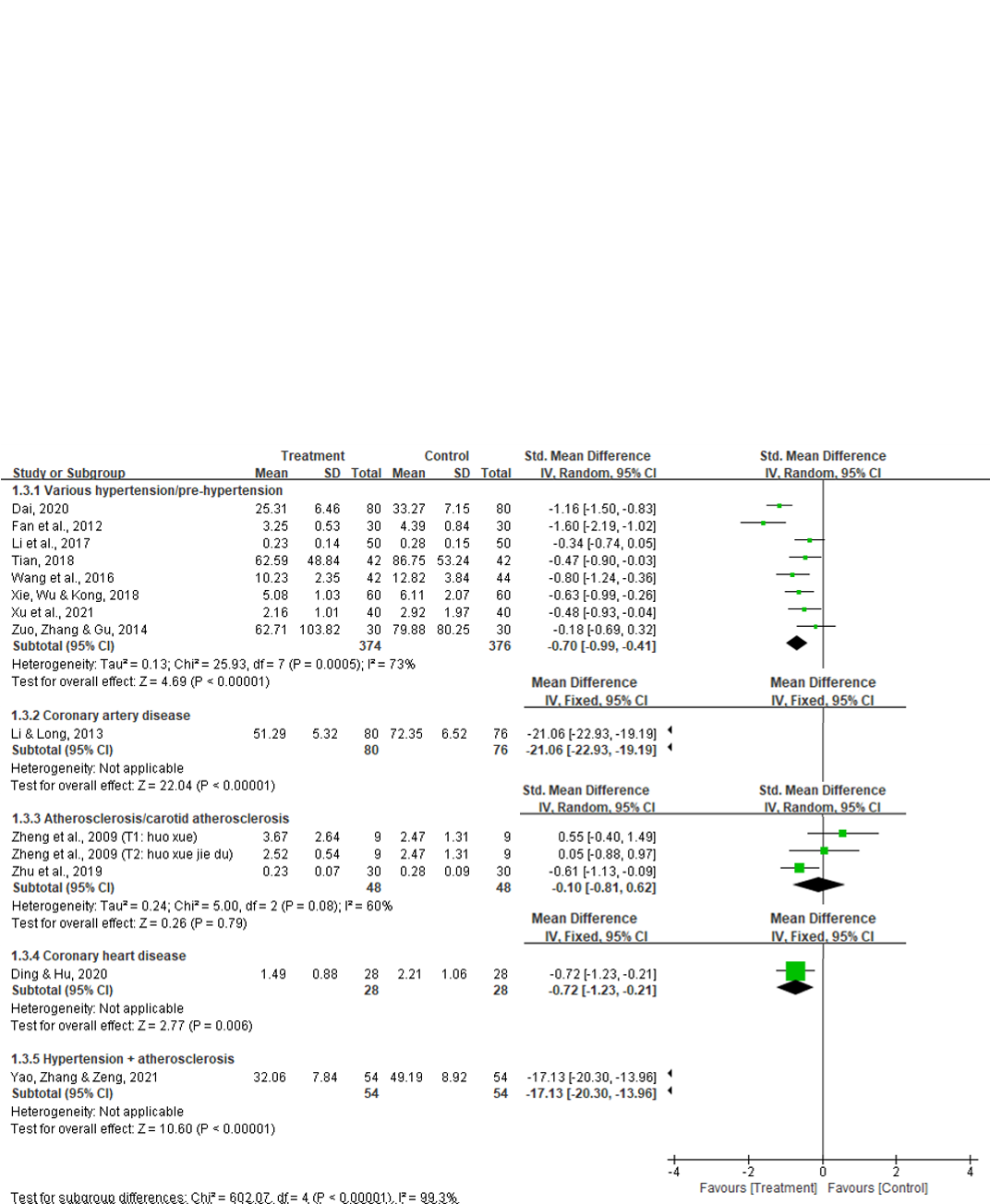
Figure S18.** Subgroup analysis of TNF-α when comparing different conditions

**
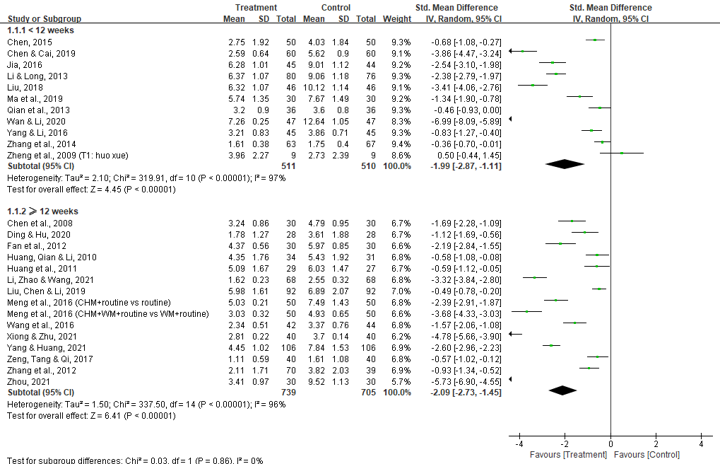
**

**Figure S19.** Subgroup analysis of hs-CRP when comparing different trial duration

**
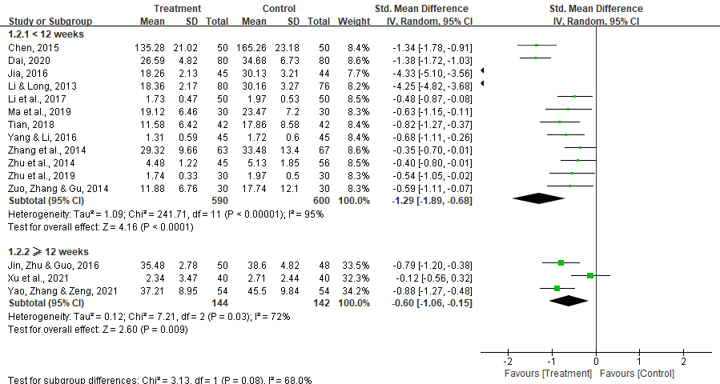
**

**Figure S20.** Subgroup analysis of IL-6 when comparing different trial duration

**
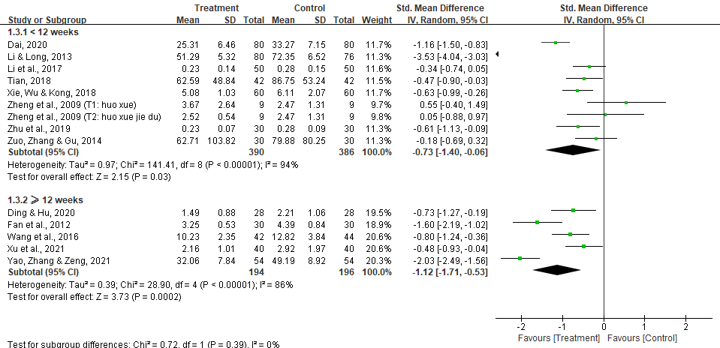
Figure S21.** Subgroup analysis of TNF-α when comparing different trial duration


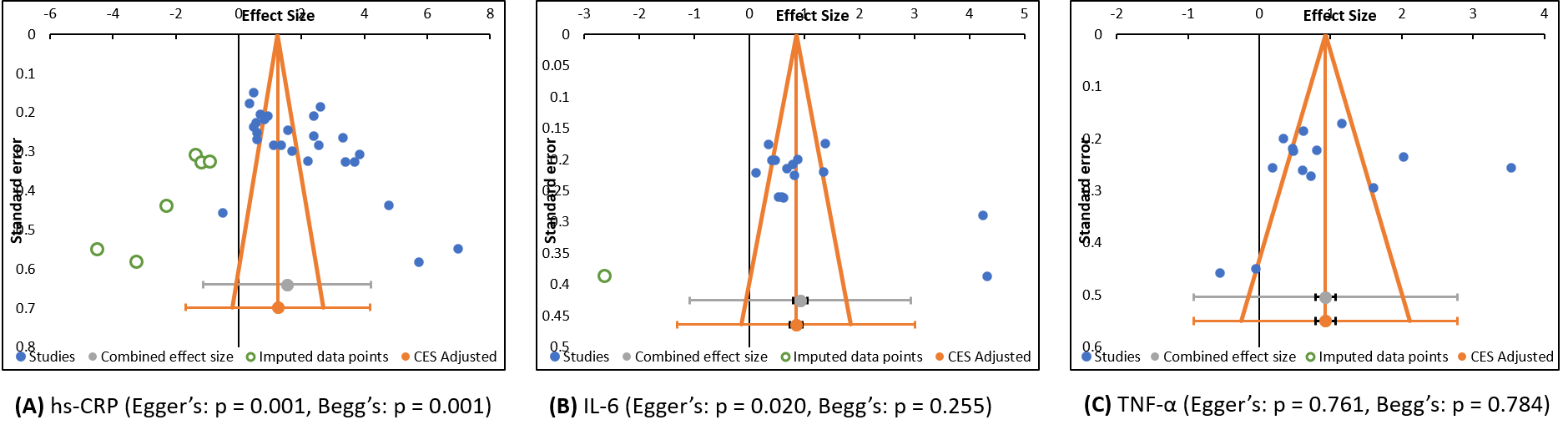


**Figure S22.** Funnel plots of change from baseline hs-CRP, IL-6 and TNF-α outcomes.
